# Ribosome phenotypes for rapid classification of antibiotic-susceptible and resistant strains of *Escherichia coli*

**DOI:** 10.1038/s42003-025-07740-6

**Published:** 2025-02-26

**Authors:** Alison Farrar, Piers Turner, Hafez El Sayyed, Conor Feehily, Stelios Chatzimichail, Sammi Ta, Derrick Crook, Monique Andersson, Sarah Oakley, Lucinda Barrett, Christoffer Nellåker, Nicole Stoesser, Achillefs Kapanidis

**Affiliations:** 1 Department of Physics, University of Oxford, Oxford, UK; 2 Kavli Institute for Nanoscience Discovery, University of Oxford, Oxford, UK; 3 School of Infection and Immunity, University of Glasgow, Glasgow, UK; 4 Nuffield Department of Medicine, University of Oxford, John Radcliffe Hospital, Oxford, UK; 5 Nuffield Department of Medicine, NIHR Health Protection Research Unit in Healthcare Associated Infections and Antimicrobial Resistance at University of Oxford in partnership with Public Health England, Oxford, UK; 6 NIHR Oxford Biomedical Research Centre, Oxford University Hospitals NHS Foundation Trust, John Radcliffe Hospital, Oxford, UK; 7 Department of Microbiology and Infectious Diseases, Oxford University Hospitals NHS Foundation Trust, Oxford, UK; 8 Nuffield Department of Women’s & Reproductive Health, University of Oxford, Big Data Institute, Oxford, UK

**Keywords:** Infectious-disease diagnostics, Bacteriology, Antimicrobial resistance

## Abstract

Rapid antibiotic susceptibility tests (ASTs) are an increasingly important part of clinical care as antimicrobial resistance (AMR) becomes more common in bacterial infections. Here, we use the spatial distribution of fluorescently labelled ribosomes to detect intracellular changes associated with antibiotic susceptibility in *E. coli* cells using a convolutional neural network (CNN). By using ribosome-targeting probes, one fluorescence image provides data for cell segmentation and susceptibility phenotyping. Using 60,382 cells from an antibiotic-susceptible laboratory strain of *E. coli*, we showed that antibiotics with different mechanisms of action result in distinct ribosome phenotypes, which can be identified by a CNN with high accuracy (99%, 98%, 95%, and 99% for ciprofloxacin, gentamicin, chloramphenicol, and carbenicillin). With 6 *E. coli* strains isolated from bloodstream infections, we used 34,205 images of ribosome phenotypes to train a CNN that could classify susceptible cells with 91% accuracy and resistant cells with 99% accuracy. Such accuracies correspond to the ability to differentiate susceptible and resistant samples with 99% confidence with just 2 cells, meaning that this method could eliminate lengthy culturing steps and could determine susceptibility with 30 min of antibiotic treatment. The ribosome phenotype method should also be able to identify phenotypes in other strains and species.

## Abstract (web summary)

Ribosome-targeting FISH probes reveal intracellular changes in response to antibiotics that can be used to train neural networks to rapidly classify *Escherichia coli* from clinical isolates as antibiotic-resistant or susceptible.

## Introduction

Bacterial infections were associated with 14% of all global deaths and the majority of sepsis-related deaths^1^ in 2019. The widespread use of antibiotics in the treatment and prevention of these infections, in medicine and in agriculture, has created a strong evolutionary pressure for microbes resistant to these compounds^2^. In 2019, antimicrobial resistance (AMR) in bacteria caused 1.27 million deaths and was associated with 4.95 million deaths worldwide^3^. Mortality is predicted to rise as high as 10 million deaths per year by 2050 if no action is taken^4^. These challenges motivate the development of new antimicrobial treatments and technologies to mitigate the effects of resistant infections.

Antibiotic susceptibility tests (ASTs) are an essential tool for refining treatment and minimising inappropriate antibiotic use. However, in most clinical microbiology pathways, ASTs are performed after a bacterial pathogen has been cultured and identified, with results available in 12–48 h for common species^5^. This time delay is often too long to wait in life-threatening infections^6^, leading clinicians to prescribe empirically and use combinations of broad-spectrum antibiotics. In clinical trials, the use of rapid ASTs improves clinical outcomes, decreases the use of broad-spectrum antibiotics, and shortens the time between sample collection and optimal targeted antibiotic treatment^7,8^.

The clinical need for rapid ASTs has motivated the development of new diagnostic technologies to identify the infecting species and characterise susceptibility. Current growth-based ASTs quantify the Minimum Inhibitory Concentration (MIC), a marker of the susceptibility of the isolated organism to an antibiotic^9^, which is typically measured using turbidity. Faster assays based on genotype and cellular morphology are being developed. The bioMerieux BioFire FilmArray system, for example, is a commercial genotype-focused platform utilising multiplex polymerase chain reaction (PCR) to detect species-specific and resistance-associated genes in syndromic infections (e.g., respiratory, bloodstream, and joint) within an hour^10^. However, PCR methods cannot detect AMR genes that are not present in the PCR probe set, and resistance genes do not always correlate with an isolate’s antibiotic response. A rapid phenotypic test that directly measures the bacterial response may offer advantages over genotypic assays, especially in Gram-negative species which are more likely to have polygenic and combinatorial mechanisms of resistance^5,11^.

Some of the discordances between the genotype and the phenotypic susceptibility may be explained by phenotypic heterogeneity within a bacterial population, leading to phenomena such as persister cells^12,13^ and viable but non-culturable cells^14^. Techniques that directly measure single-cell antibiotic response are advantageous because they can capture this heterogeneity. Many methods have been proposed, including using microscopy to measure growth rate^15,16^, antibiotic accumulation^17^, structural changes^18,19^, filamentation^20^, or cell death^21^; flow cytometry^22^; Raman spectroscopy^23^; cell impedance^24^; elastic light scattering^25^; and nanomotion classification^26^. An example of a commercially available phenotypic system is the Accelerate Diagnostics Pheno System, which combines fluorescence in situ hybridisation (FISH) for species identification with monitoring of single-cell growth rates to report antibiotic resistance within 7 h^27^.

Visually apparent changes to the intracellular structure of the bacterial cells can also be used to measure the bacterial antibiotic response. When antibiotics disrupt cellular physiology, long-recognised and characteristic phenotypes develop, which have recently been characterised at scale with high-content imaging^28,29^. Our group showed that such phenotypic effects on the nucleoid and cell membrane can be visualised within 30 min and recognised by trained deep-learning models, and that this variability correlates with clinical antibiotic susceptibility^30^. While many novel ASTs have been proposed and developed^5,15,16,18,19,21–27^, by using single-cell imaging data, we can rapidly and directly capture and assay the diversity of antibiotic response within the cell population.

**Fig. 1:**
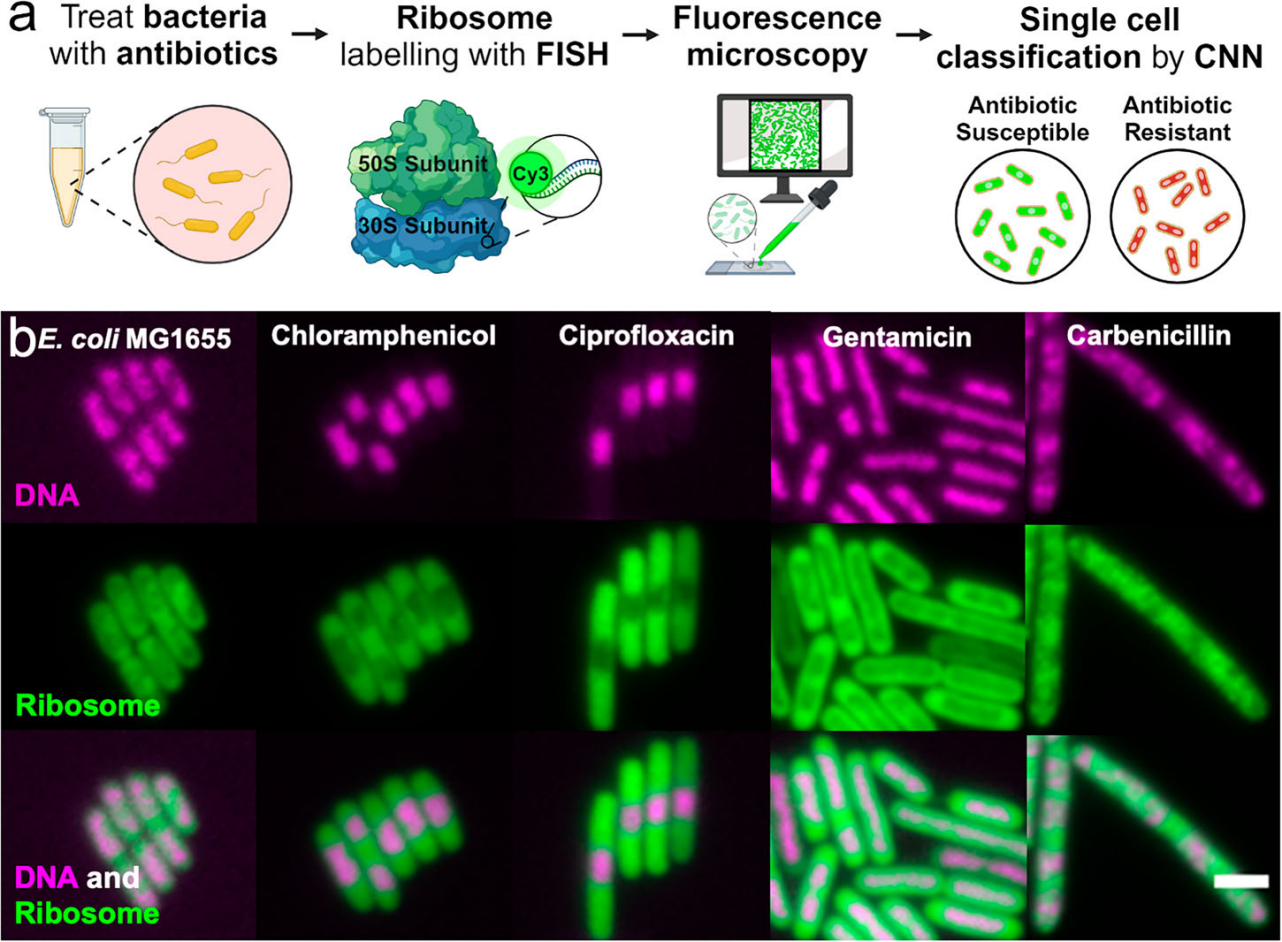
Proposed workflow for using ribosome phenotypes in a rapid antibiotic susceptibility test. **a** Workflow: First the clinical isolate is treated with antibiotics at a standard concentration for 30 min. Then, a standard FISH protocol is used to label the ribosomes with ssDNA fluorescent probes; in this case, EUB338-Cy3 binds a conserved region in the 30S subunit. The samples are imaged on a fluorescence microscope before neural networks use the ribosome signal to segment and then classify the cells as susceptible or resistant to the prescribed antibiotic treatment. **b** Ribosome Phenotypes: Representative fluorescence images are shown of *E. coli* MG1655 with and without antibiotic treatment (magenta, DNA stained with DAPI; green, ribosomes labelled with EUB338-Cy3 probes; combined DNA and ribosome signal). The scale bar is 2 µm. The ribosome density can be seen to anti-correlate with the DNA-dense regions. The untreated panel shows fixed cells with no antibiotic treatment. The chloramphenicol panel shows cells treated with 8 mg/L chloramphenicol (1 × EUCAST breakpoint) for 30 min before fixation. The ciprofloxacin panel shows the same, treated with 0.5 mg/L ciprofloxacin (1 × EUCAST breakpoint). The gentamicin panel shows the same, treated with 40 mg/L gentamicin (20 × EUCAST breakpoint). The carbenicillin panel shows the same, treated with 24 mg/L carbenicillin (3 × EUCAST breakpoint).

Here, we present a method for rapid identification of single-cell antibiotic susceptibility by detecting intracellular changes using ribosome-bound FISH probes (Fig. 1a). First, bacteria from the clinical sample are treated with an antibiotic that will induce phenotypic changes in bacteria susceptible to the antibiotic. Following the antibiotic treatment, the cells are fixed, permeabilised, and incubated with species-specific ribosome-targeting FISH probes. This protocol takes approximately 2 h, and can still be further optimised for speed.

Because of the spatial anti-correlation between DNA and ribosome density within the cell^31^, we reasoned that an image of a cell’s ribosome distribution could be used like an inverse DNA stain to visualise structural changes associated with the antibiotic response. We call this distribution the ribosome phenotype. Images of the ribosome phenotypes are processed and fed to a pre-trained neural network to classify the bacteria as antibiotic-susceptible or antibiotic-resistant.

The antibiotic treatment concentrations were chosen as a multiple of the European Committee on Antimicrobial Susceptibility Testing (EUCAST) breakpoint^9^ for *Enterobacterales* including *E. coli*, so that an empirical benchmark could be applied to other strains. The EUCAST breakpoint is a defined concentration of antibiotic used to classify a microorganism as antibiotic susceptible or resistant, accounting for clinical factors including antibiotic dosage, target infections, pharmacokinetics, and resistance mechanisms. The MIC of a bacterial isolate can be compared to the EUCAST breakpoint for a given antibiotic and bacterial species to classify it as clinically susceptible (S) or resistant (R). The antibiotic concentration should be optimised depending on the antibiotic’s mechanism of action to induce differentiable phenotypes in susceptible and resistant strains within the 30-min treatment period.

We found that a convolutional neural network (CNN) could learn to recognise the distinct ribosome phenotypes of *E. coli* treated with antibiotics from four classes used to treat *E*. coli infections with different mechanisms of action (ciprofloxacin, a fluoroquinolone, targeting DNA gyrase/topoisomerases; gentamicin, an aminoglycoside, targeting the 30S ribosomal subunit; chloramphenicol, an amphenicol, targeting the 50S ribosomal subunit; and carbenicillin, a β-lactam targeting peptidoglycan synthesis). We applied the ciprofloxacin CNN to classify three ciprofloxacin-susceptible and three ciprofloxacin-resistant *E. coli* clinical isolates and found that its accuracy decreased when the isolate’s antibiotic response diverged from the lab strain’s phenotype. Therefore, we developed a CNN trained on images of the clinical isolates, which was able to classify unseen, holdout single-cell images as antibiotic-susceptible with >90% accuracy and antibiotic-resistant with >99% accuracy based on their ribosome phenotypes. Our method advances existing phenotypic ASTs because, when used in combination with multiplexed FISH and species-specific probes, the ribosome fluorescent profile can be used to segment single cells, identify bacterial species, and characterise a cell’s antibiotic response in a single step.

## Results

### Characterisation of the *E. coli* antibiotic response by ribosome subcellular distribution

To train a machine-learning model to classify antibiotic-resistant and antibiotic-susceptible *E. coli*, we characterised the antibiotic response phenotypes of antibiotic-susceptible cells. Previous work has shown the successful classification of antibiotic-susceptible and resistant *E. coli* by a CNN trained to identify changes in DNA morphology^30^. It has also been shown that DNA-rich and ribosome-rich regions spatially anti-correlate in *E. coli*^31^. Therefore, we reasoned that we may be able to use ribosome phenotypes to classify a bacterium’s antimicrobial response. Because of their space-filling properties, we also hypothesised that the ribosome signal should suffice for both cell segmentation and phenotype analysis, eliminating the need for a membrane dye for cell segmentation. Ribosome fluorescence images may also provide richer spatial and intensity features throughout the cell than images of the nucleoid morphology.

To test our hypothesis, we first characterised the sub-cellular ribosome phenotypes of antibiotic-susceptible *E. coli* MG1655 (Fig. 1b). MG1655 is a lab-adapted K-12 derivative that is susceptible to each of the antibiotics used in this work. After treatment with each of the 4 antibiotics individually for 30 min, the cells were stained with fluorescent FISH probes to visualise the effects of antibiotic treatment on their internal structure. For this, we used an 18-mer single-strand DNA probe with Cy3 dye conjugated to the EUB338 sequence, which targets a region in the 16S ribosomal RNA conserved in all members of the domain *Bacteria*^32^.

The biological effect of antibiotic treatment on the susceptible MG1655 bacteria can be clearly seen within 30 min. Fluorescence images of the DNA and ribosomes show the characteristic changes in cell spatial organisation that occur as the cell responds to the antibiotic (Fig. 1b). Comparing the DNA and ribosome signals shows the anti-correlation between DNA and ribosome density within the cell in untreated and antibiotic-treated conditions. The nucleoid compaction caused by chloramphenicol, ciprofloxacin, and gentamicin can be seen as clearly in the ribosome images as in the DNA images. For carbenicillin, the cell filamentation and multiple DNA regions with lower ribosome intensity can be seen. These images also show how the ribosomes fill the cell, allowing the ribosome signal to be used for both cell phenotyping and cell segmentation.

We characterised the ribosome phenotypes from four biological replicates of *E. coli* MG1655 totalling 5286 untreated cells, 3215 cells treated with ciprofloxacin (Cip) at 0.5 mg/L (1 × EUCAST breakpoint), 5935 cells treated with gentamicin (Gent) at 40 mg/L (20 × EUCAST breakpoint), 6439 cells treated with chloramphenicol (Cam) at 8 mg/L (1 × EUCAST breakpoint), and 1256 cells treated with carbenicillin (Carb) at 24 mg/L (3 × EUCAST breakpoint). For *E*. coli MG1655, treatment with chloramphenicol or ciprofloxacin at 1 × EUCAST induced phenotypic changes within 30 min, but gentamicin treatment concentrations lower than 20 × EUCAST and carbenicillin treatment concentrations lower than 3 × EUCAST did not induce phenotypic changes in most cells in this time frame (Figs. S1, S2). These results for gentamicin and carbenicillin suggest that the antibiotic treatment concentration should be optimised for each antibiotic and its mechanism of action. This optimal treatment concentration can serve as a benchmark to characterise the magnitude of response in other strains.

**Fig. 2:**
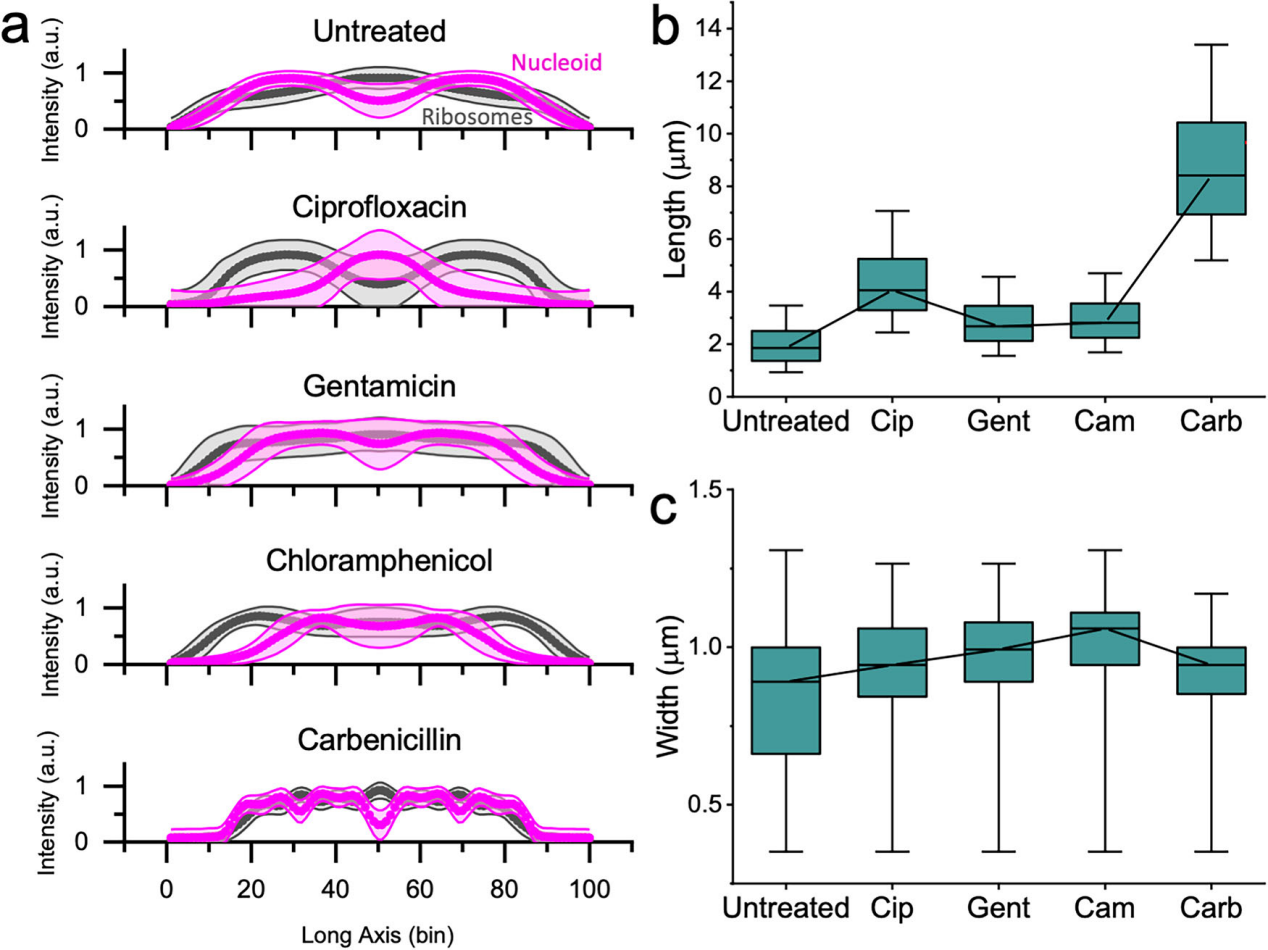
Ribosome intensity line profiles reveal ribosome-nucleoid anti-correlation and characteristic phenotypes of antibiotic response. **a** Along the long axis of the cell, the mean normalised ribosome (Cy3, grey) and nucleoid (DAPI, magenta) intensities are calculated for each of 100 bins. The shading shows ± 1 standard deviation of the mean. The untreated *E. coli* line profiles show two nucleoid-rich regions, correlated with decreased ribosome intensity. This figure is composed of profiles from untreated *E. coli* MG1655 (*N* = 5286). The ciprofloxacin panel shows the same, for *E. coli* MG1655 treated with 1 × EUCAST ciprofloxacin for 30 min (*N* = 3215). The line profile shows a central, compact nucleoid region with greater segregation from the ribosomes. The gentamicin panel shows the same, for *E. coli* MG1655 treated with 20 × EUCAST gentamicin for 30 min (*N* = 5935). This line profile shows a diffuse nucleoid region along the long axis of the cell with less ribosome-nucleoid segregation. The chloramphenicol panel shows the same, for *E. coli* MG1655 treated with 1 × EUCAST chloramphenicol (*N* = 6438). This line profile shows nucleoid compaction compared to the untreated phenotype, with a centralised DNA region or two dense DNA regions. The carbenicillin panel shows the same, for *E. coli* MG1655 treated with 3 × EUCAST carbenicillin (*N* = 1256). This line profile shows ~4 regions of DNA density, indicating that DNA replication and cell growth has continued without cell division. **b** The cell lengths (μm) are shown for untreated *E. coli* MG1655 and for each of the antibiotic treatments. The box shows the 25–75% percentile range, the bars show the 1–99% percentile range, and the line denotes the median. Each antibiotic results in a length distribution statistically different from the untreated population with *p* < 0.05 by the Mann-Whitney non-parametric test. **c** The cell widths (μm) are shown for untreated *E. coli* MG1655 and for each of the antibiotic treatments. The box shows the 25–75% percentile range, the bars show the 1–99% percentile range, and the line denotes the median. Each antibiotic results in a width distribution statistically different from the untreated population with *p* < 0.05 by the Mann-Whitney non-parametric test.

By inspecting the nucleoid and ribosome fluorescence signals along the long axis of the cells, we can further characterise the treatment phenotypes. In untreated *E. coli*, the highest ribosome density was seen in the centre of the cell and in longer cells there were often two ribosome-poor nucleoid regions (Figs. 1b; 2a). Ciprofloxacin treatment caused a central, compact nucleoid region (Figs. 1b; 2a) and resulted in cells that were longer than untreated cells (Fig. 2b). Gentamicin treatment led to a diffuse nucleoid region following the long axis of the cell that was often rod-shaped (Figs. 1b; 2a). Chloramphenicol treatment caused nucleoid compaction compared to the untreated phenotype, causing either a centralised DNA region or two dense DNA regions (Figs. 1b; 2a). Carbenicillin treatment caused filamentation with much longer cells (Fig. 2b) and four copies of the DNA in the typical cell (Figs. 1b; 2a). All antibiotic treatments resulted in cells with significantly different in average length and average width compared to the untreated phenotype (Mann-Whitney non-parametric hypothesis test *p* < 0.05) (Fig. 2b, c). The antibiotic treatment phenotypes in our images aligned with those found in previous work^33–35^ and with the mechanism of action of each antibiotic (Figs. 1b; 2). In the case of ciprofloxacin and carbenicillin, the changes in cell length and morphology may be sufficient to judge an antibiotic response^19^, but ribosome phenotypes provide data on internal changes. Because these phenotypes are quantifiable by ribosome fluorescence intensity mapping and identifiable by the human eye, it follows that a neural network could be trained to associate them with an antibiotic treatment response.

### Antibiotic-susceptible ribosome phenotypes are identified accurately by a neural network

To train neural networks that can robustly identify the ribosome phenotypes resulting from antibiotic treatments, our fluorescence images were pre-processed prior to their use as training data. First, each single-channel image was segmented by a custom CellPose^36^ model trained to segment *E. coli* by ribosome fluorescence profiles. The segmentations were subsequently curated to refine the outlines and remove cells that were outside of the field of view, overlapping, or outside of the focal plane^37^. To regularise learning and prevent overfitting, each segmentation was used to create a 64 × 64 zero-filled image with the ribosome fluorescence in the centre, and augmentations (e.g., brightness normalisation, random noise, and geometric transformations) were applied before each image was loaded into the training dataset (Fig. S3). We tried augmentation strategies with and without shearing and blurring, which could cause distortions of the ribosome phenotype and hinder learning. The mean balanced accuracy of the gentamicin and chloramphenicol models improved when shearing and blurring was removed (Fig. S4). For CNN training, long cells need to be resized or cropped to a standard size (64 × 64). We trained models on cropped cells and on resized cells with a mantained aspect ratio. Remarkably, resizing or cropping changed the accuracy of the ciprofloxacin and carbenicillin models by less than 0.1%; we thus used the cropping method to preserve the intracellular definition (Fig. S5).

**Fig. 3:**
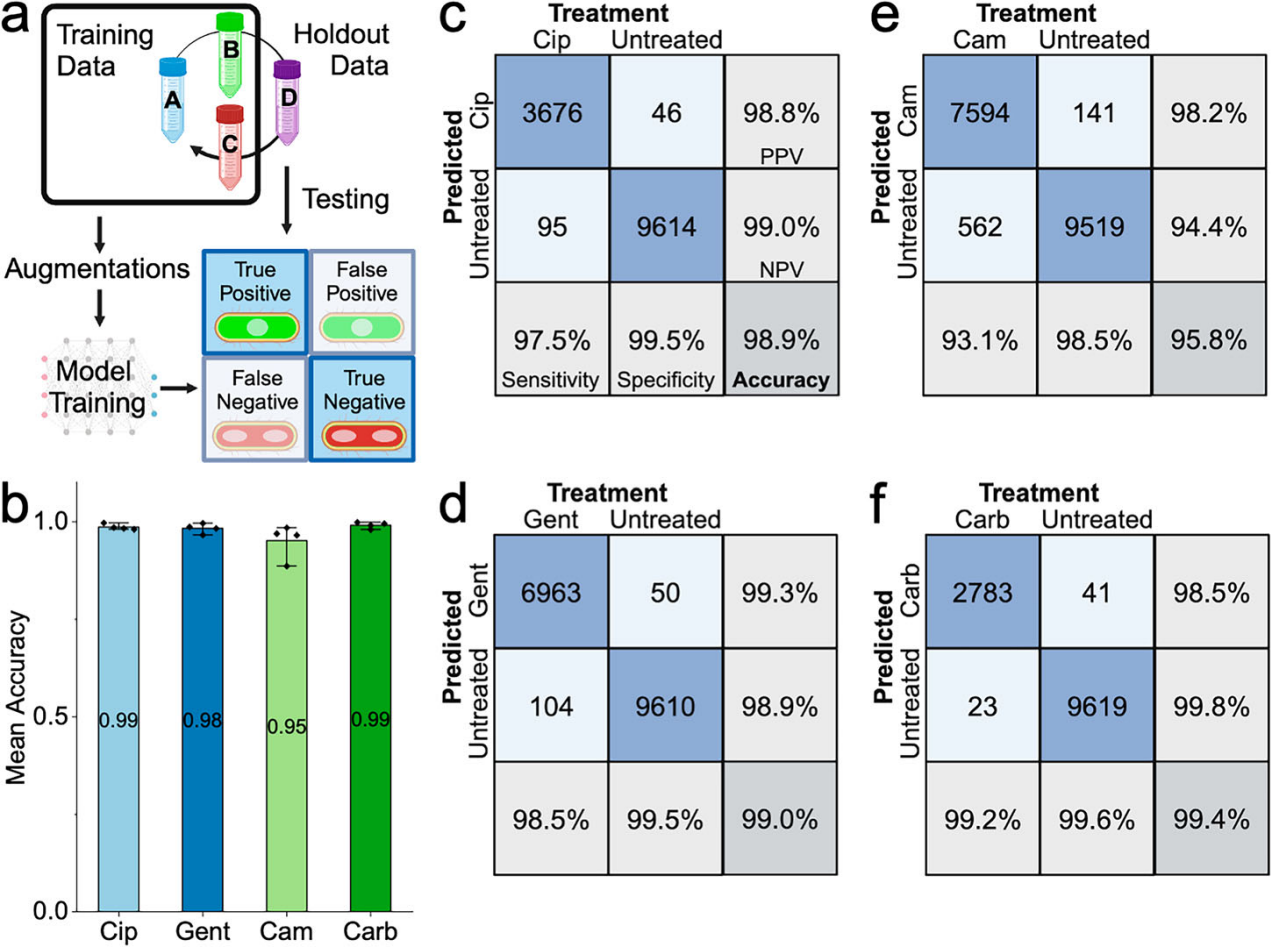
Ribosome phenotype recognition is robust across biological replicates. **a** Four biological replicates of *E. coli* MG1655 were tested for each antibiotic and for the untreated condition. To test phenotype robustness and repeatability, a holdout cross-validation was performed in which each model was trained and validated on images from three of the biological replicates and tested on images from the fourth. The training images received random data augmentations before being passed to the model, whereas the holdout dataset was passed directly to the model for testing. **b** The balanced accuracy of the ribosome phenotype classifier is shown for each antibiotic. Each point represents a biological replicate. The mean balanced accuracy is shown on each column and the error bars indicate the 95% confidence interval of the mean on the four biological replicates. **c** Confusion matrices for the ciprofloxacin (Cip) ribosome phenotype classifier. The total number of cells is a sum of the results from four experiments, each with a model trained on three biological replicates and tested on a fourth holdout replicate. The number of images in each class is shown, along with the percentage of cells for each treatment condition. The treatment condition is shown on the columns and the model’s predicted classification is shown on the rows. Right column: positive predictive value (PPV) and negative predictive value (NPV) of the model’s predictions are shown. Bottom row: accuracy of the model on antibiotic-treated cells (Sensitivity), accuracy of the model on untreated cells (Specificity), and the Balanced Accuracy (Accuracy) are shown. See *Accuracy Metrics* for details. **d** As in (**c**), for the gentamicin (Gent) ribosome phenotype classifier. **e** As in (**c**), for the chloramphenicol (Cam) ribosome phenotype classifier. **f** As in (**c**), for the carbenicillin (Carb) ribosome phenotype classifier.

To test the reliability and accuracy of the neural network in differentiating the antibiotic-susceptible phenotypes from untreated *E. coli* MG1655, a rotating holdout test was performed (Fig. 3a). For each experiment, a model was independently trained and validated on data from three of the biological replicates and tested on the fourth. The validation and testing datasets were balanced to include an equal number of untreated and antibiotic-treated cell images to minimise prediction biases. The average balanced accuracy of the models on the four test datasets exceeded 95% for all antibiotics: ciprofloxacin~99 ± 1.5%, gentamicin~98 ± 2.5%, chloramphenicol~95 ± 8.8%, carbenicillin~99 ± 1.6% (Fig. 3b). Looking at the models’ predictions, the cells that were classified with high confidence (Fig. S6) demonstrated the characteristic antibiotic phenotypes (Fig. 2).

We then examined the confusion matrices for the four holdout datasets, summed together. The ciprofloxacin phenotype model was one of the most accurate, with a very high average balanced accuracy of 98.9%, sensitivity of 97.5%, and specificity of 99.5% (Fig. 3c). This may be because the ciprofloxacin response causes two phenotypic changes – elongated cells and condensed, central nucleoid – both of which can be used by the model in the classification task (Fig. S7). The gentamicin phenotype model was also highly accurate, achieving an average balanced accuracy of 99.0%, sensitivity of 98.5%, and specificity of 99.5% (Fig. 3d). The chloramphenicol phenotype model had a balanced accuracy of 95.8%, sensitivity of 93.1%, and specificity of 98.5% (Fig. 3e). Inspection of the chloramphenicol-treated cells that were misclassified as untreated suggests that this model’s increased number of False Negative classifications was driven by cells that did not adopt the expected chloramphenicol-treated phenotype within the antibiotic treatment period, having multiple, diffuse nucleoid regions (Fig. S8a), whereas False Positive classifications tended to have a central nucleoid region (Fig. S8b). Finally, the carbenicillin phenotype model had a balanced accuracy of 99.4%, sensitivity of 99.2%, and specificity of 99.6% (Fig. 3f). The balanced accuracies of >95% reported here are for single cells and therefore the cumulative accuracy of the classifier on a collection of cells is essentially 100%. Accuracy on a sample of cells is discussed further in the section on clinical isolates.

### Ribosome phenotypes can be used to classify ciprofloxacin-resistant clinical *E. coli* isolates

Having demonstrated that antibiotic response phenotypes can be reliably induced and classified by a CNN, we moved to train a model to classify *E. coli* isolated from clinical samples as susceptible or resistant to ciprofloxacin using the ribosome phenotype. We called an isolate “resistant” if its minimum inhibitory concentration (MIC, the concentration required to inhibit overnight growth), was above the EUCAST breakpoint^9^. Isolates with MICs below the EUCAST breakpoint were called “susceptible”. We hypothesised that a CNN could learn to identify ribosome phenotypes associated with ciprofloxacin sensitivity or resistance, and that resistant cells would look similar to untreated cells^30^ following ciprofloxacin exposure.

To represent some of the variation present in pathogenic *E. coli*, we chose three susceptible strains (S1, S2, S3) and three resistant strains (R1, R2, R3), each with a different mutation in ciprofloxacin resistance-associated genes (Table 1). Each of the susceptible strains have a mutation in one of these genes, whereas the resistant strains all have three or more resistance-associated mutations. For example, strain R2 has two mutations in the *GyrA*, which encodes the A subunit of DNA gyrase; and three mutations in the genes that encode DNA topoisomerase IV (two mutations in *parC*, for the A subunit; and one in *parE*, for the B subunit). Each of these mutations has been associated with increased ciprofloxacin resistance in previous work^38–41^. Each isolate was treated with ciprofloxacin at a concentration previously determined to robustly induce phenotypic changes within 30 minutes (10 mg/L, 20 × EUCAST breakpoint)^30^.

**Table 1:** *E. coli* clinical isolates with their MICs and AMR genotypes

*E. coli* Clinical Isolate	Ciprofloxacin MIC (mg/L)	Relevant genotype information			
		*gyrA*	*marR*	*parC*	*parE*
Susceptible 1 (S1)	0.015				I355T
Susceptible 2 (S2)	0.25		S3N		
Susceptible 3 (S3)	0.25				I529L
Resistant 1 (R1)	2	D87Y, S83L		S80I	
Resistant 2 (R2)	16	D87N, S83L		E84V, S80I	I529L
Resistant 3 (R3)	64	D87N, S83L		S80I	S458A
Resistant 4 (R4)	128	D87N, S83L		S80I	S458A

Each clinical isolate used in this project is listed with its MIC and relevant genotype information. All strains are *Escherichia coli* isolated from bloodstream infections in the United Kingdom, obtained and whole-genome sequenced for a previous study^57^. MICs were determined by broth microdilution. (See *Methods: Bacterial strains and sample preparation* for details of MIC and sequencing methods).

**Fig. 4:**
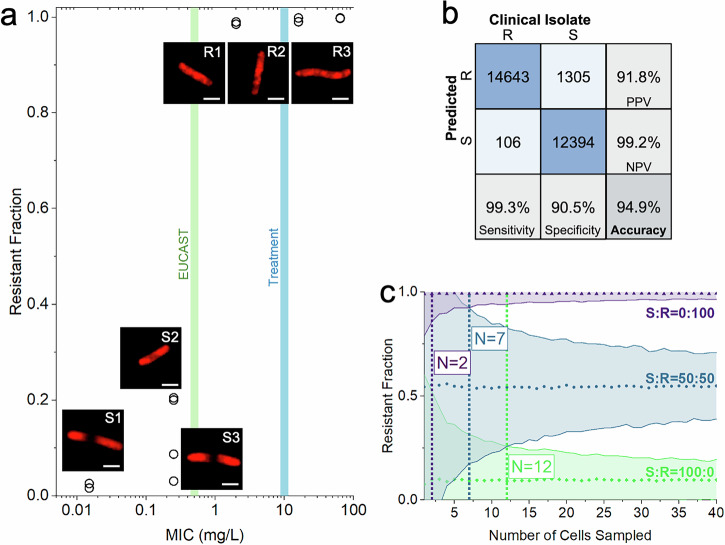
Susceptible (MIC < EUCAST breakpoint) and resistant (MIC > EUCAST breakpoint) *E. coli* isolates can be differentiated by the fraction of cells called resistant by the model. **a** The fraction of cells in the sample called resistant by the susceptible-resistant classifier (Resistant Fraction) is plotted against the MIC of the strain (mg/L) on a logarithmic scale, with each biological replicate represented as a circle. The test dataset is composed of holdout images, previously unseen by the classifier, from each clinical isolate. The EUCAST breakpoint (0.5 mg/L, green) and the treatment condition (10 mg/L, blue) are shown with shaded vertical lines. All strains with an MIC below the EUCAST breakpoint have a resistant fraction less than or equal to 0.2, whereas the fraction classified resistant is nearly 1.00 for the strains with an MIC above the EUCAST breakpoint. Representative, correctly classified images of ribosome phenotypes from each of the clinical isolates are shown for each point. Scale bar, 2 μm. **b** The confusion matrix for the ciprofloxacin-resistant and ciprofloxacin-susceptible classifier trained on 6 strains on a holdout, unseen dataset of those 6 strains.The testing dataset is composed of 28,448 images from unseen biological replicates. See *Accuracy Metrics* for details on Accuracy, Sensitivity, Specificity, PPV, and NPV. **c** The number of cells necessary to classify a sample as coming from a population of susceptible or resistant bacteria with 99% confidence. Simulated samples of different susceptible:resistant ratios (S:R) were transformed through the sensitivity and specificity of the susceptible-resistant classifier to determine the minimum number of cells necessary to differentiate them. Here, we plot the mean Resistant Fraction and 99% confidence interval after 1000 trials with samples ranging from 1 to 40 cells sampled (N) for susceptible:resistant ratios of 0:100 (purple triangles), 50:50 (blue circles), and 100:0 (green diamonds). As the number of cells sampled increases, the confidence interval of the Resistant Fraction narrows. Susceptible samples can be differentiated from resistant samples with a sample of 2 cells (purple dotted line). A mixed sample can be differentiated from a resistant sample with 7 cells (blue dotted line) or from a susceptible sample with 12 cells (green dotted line). The confidence interval for resistant cells is narrower than that of susceptible cells because the classifier is more sensitive than it is specific.

Following ciprofloxacin treatment, all susceptible *E. coli* strains (S1, S2, & S3) demonstrated a ribosome phenotype similar to ciprofloxacin-treated *E. coli* MG1655, with a compact, central nucleoid region that we can detect indirectly because it results in low ribosome density in the central region (Figs. 4a: S1, S2, S3). Some also showed an elongated morphology (Figs. 4a: S1, S3). While some cells from the resistant strains resembled untreated *E. coli* MG1655, most cells from the resistant strains showed a different ciprofloxacin response, wherein the cells were elongated but retained diffuse nucleoid regions that spanned the cell length (Fig. 4a: R1, R2, R3).

In previous work, we have shown that a classifier trained on the nucleoid phenotypes of untreated and ciprofloxacin-treated *E. coli* MG1655 was able to classify susceptible and resistant clinical isolates accurately because resistant clinical isolates resembled untreated MG1655, while susceptible clinical isolates resembled ciprofloxacin-treated MG1655^30^. We applied this method to our ribosome images, using the CNN trained on ciprofloxacin-treated MG1655 to classify clinical isolates.

The MG1655 ciprofloxacin classifier had variable accuracy when applied to ciprofloxacin-treated clinical isolates (Fig. S9). The classifier recognised ciprofloxacin-susceptible phenotypes with high accuracy. For S1 and S3, it classified cells as ciprofloxacin-susceptible with 99.0 ± 1.0% and 95.4 ± 4.2% accuracy, respectively. The accuracy was lower for S2 cells (81.2 ± 0.5%), possibly because these cells are less likely to be elongated than ciprofloxacin-treated MG1655 (Mann-Whitney non-parametric hypothesis test *p* < 0.05; Figs. 4a, S2 isolate). For resistant isolates, the MG1655 classifier was less reliable. Compared to untreated MG1655, the ciprofloxacin-treated resistant isolates had similarly diffuse nucleoid regions but were elongated (Fig. 4a: R1, R2, R3). The MG1655 classifier classified 95.2 ± 5.8% of R2 cells as ciprofloxacin-resistant, but only 54.0 ± 3.7% of R1 cells and 74.3 ± 1.9% of R3 cells (Fig. S9b). Representative cell images show that the R1 cells that were misclassified as susceptible had an elongated cell shape and a diffuse nucleoid (Fig. S6a: R1), whereas R2 cells that were correctly classified as resistant had a shape and nucleoid phenotype more similar to the untreated MG1655 (Fig. S6a, R2 isolate). The variability in accuracies for R1, R2, and R3 clearly show that the ribosome phenotypes resulting from 20 × EUCAST ciprofloxacin treatment are too diverse to be reliably recognised by the MG1655 classifier, especially for resistant strains that develop an elongated shape with a diffuse nucleoid.

Therefore, we hypothesised that a CNN trained on images of ciprofloxacin-susceptible and resistant clinical *E. coli* isolates would be able to learn these variable responses and would perform better at the classification task. For this model, the training dataset was composed of 34,205 *E. coli* cells from clinical isolates treated with ciprofloxacin, which were segmented, zero-filled, and augmented as was done for the *E. coli* MG1655 model. We trained two six-strain models to check consistency on different biological replicates. For each of the six-strain models, two biological replicates were used for the training and validation datasets and one was used for a holdout test to assess the model’s accuracy on unseen data. In total, the testing dataset comprised 28,448 cells from three susceptible and three resistant clinical isolates (Fig. 4b).

The susceptible-resistant CNN learned to identify phenotypes associated with ciprofloxacin-treated susceptible and resistant strains with a single-cell balanced accuracy of 95.0 ± 0.3%; across all strains, it displayed an accuracy of 99.3 ± 0.2% in classifying resistant cells and an accuracy of 90.5 ± 0.5% in classifying susceptible cells (Fig. 4b). Because these high accuracies were on a per-cell basis, we were able to estimate the power of the model to classify an unknown population of *E. coli* as antibiotic-susceptible, antibiotic-resistant, or a mixture of the two. We simulated cell samples with 100% resistant cells, 100% susceptible cells, or a 50-50 mixture, using the sensitivity and specificity of our assay.

Given its high sensitivity (99.3%) and specificity (90.5%), our susceptible-resistant classifier has the power to differentiate a 100% resistant sample from a 100% susceptible sample with 99% confidence after sampling as few as 2 cells (Fig. 4c). With a reasonable sample size of 10-100 bacteria isolated from a clinical specimen, the confidence level of our prediction would increase. In the case of a mixed infection or contaminated sample, we could differentiate a mixed sample from a resistant sample (7 cells) or a susceptible sample (12 cells) with the same level of confidence (Fig. 4c). Stratifying the classifications by strain, we showed that our classification accuracy remained greater than or equal to 80% for all six strains (Fig. S10). When examining the relationship between a given strain’s MIC and the fraction of cells classified as resistant, fewer than 20% of cells from strains with an MIC less than the EUCAST breakpoint were called resistant, whereas nearly 100% of cells from strains with an MIC above the EUCAST breakpoint were called resistant (Fig. 4a). Compared to the MG1655 model, the susceptible-resistant model has similar or higher accuracy for all susceptible strains and is more accurate on all resistant strains ( + 44.8 ± 4.5% for R1, +4.3 ± 1.6% for R2, +25.5 ± 2.1% for R3) (Fig. S11).

### Ribosome phenotypes can be used to classify unseen strains and antibiotic concentrations

**Fig. 5:**
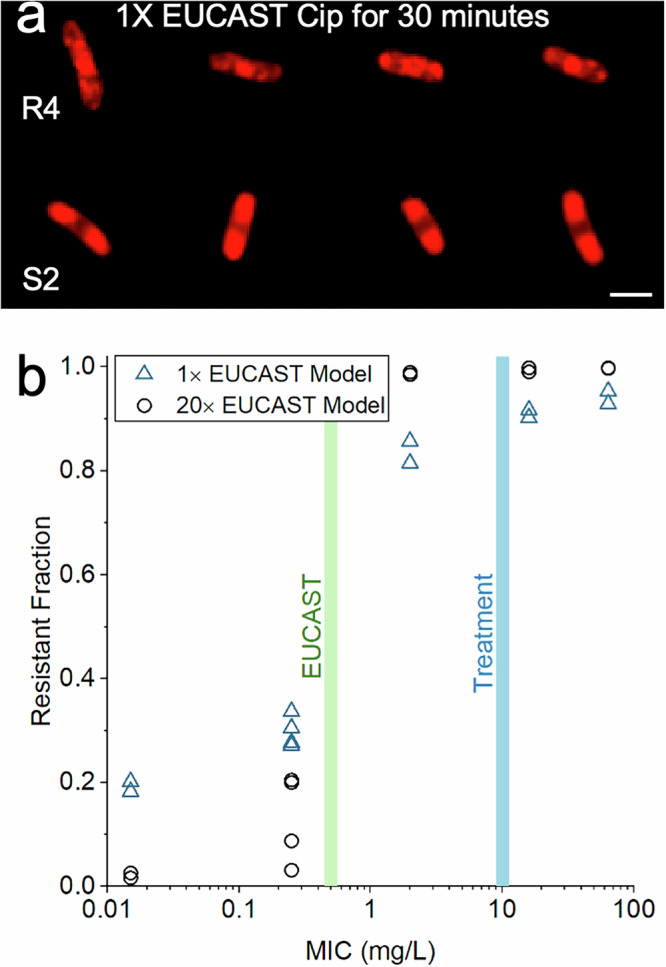
Model performs with >80% accuracy when trained on cells treated with 1 × EUCAST ciprofloxacin and tested on cells treated with 20 × EUCAST. **a** Representative, correctly classified images of the ribosome phenotypes of strains R4 and S2 treated at 1 × EUCAST (0.5 mg/L) for 30 min. Scale bar, 2 μm. **b** The susceptible-resistant classifier trained on *E. coli* treated with 20 × EUCAST ciprofloxacin (black circles) is compared to the classifier trained on *E. coli* treated at 1 × EUCAST (blue triangles). The 20 × EUCAST dataset is composed of 6 clinical isolates (S1, S2, S3, R1, R2, R3) whereas the 1 × EUCAST dataset is composed of 2 clinical isolates (S2, R4). Each data point represents a biological replicate. The 20 × EUCAST model was tested on 28,448 holdout test images from 2 biological replicates of the 6 clinical *E. coli* isolates treated at 20 × EUCAST ciprofloxacin for 30 min. The 1 × EUCAST model was tested on the same 20 × EUCAST dataset. For every isolate, the 20 × EUCAST model is more likely to call resistant cells resistant and less likely to call susceptible cells resistant. However, the 1 × EUCAST model maintains an accuracy of 73.8 ± 5.3% on cells from susceptible strains and an accuracy of 89.6 ± 3.8% on cells from resistant strains, despite being trained on images of cells treated at a different concentration and classifying a previously unseen strain (R4).

We then explored the generalisability of a CNN to previously unseen strains and antibiotic concentrations. We trained another model on just one susceptible (S2) and one resistant strain (R4) treated with 1 × EUCAST ciprofloxacin (0.5 mg/L) for 30 min (Fig. 5a). This model was trained on a dataset of 2888 cells with an 80:20 training-validation split and tested on the same holdout dataset as the six-strain model, composed of unseen cells from the six clinical isolates treated at 20 × EUCAST (10 mg/L) for 30 min. The 1 × EUCAST model was tested on each of the 3 biological replicates and performed with an average accuracy of 73.8 ± 5.3% on cells from susceptible strains and an accuracy of 89.6 ± 3.8% on cells from resistant strains (Fig. 5b). Although lower in accuracy than the six-strain model, the two-strain 1 × EUCAST model demonstrates an ability to reliably differentiate susceptible and resistant cells with relatively high accuracy while classifying cells from never-before-seen strains treated at a different concentration of ciprofloxacin. The model’s ability to generalise on unseen clinical isolates treated at a different antibiotic concentration demonstrates the robustness of the ribosome phenotype classification method, so long as the model has seen sufficiently similar training data. When the 20 × EUCAST clinical isolate classifier was applied to the 1 × EUCAST isolates, it performed well when classifying R4 (93.0 ± 12.3%), which had a similar phenotype to the other resistant strains, but was unable to reliably classify S2 (52.3 ± 11.0%), which was much less elongated than the susceptible strains treated at higher antibiotic concentrations and had a less defined nucleoid region (Fig. S12).

### Simultaneous identification of bacterial species and ribosome phenotype

**Fig. 6:**
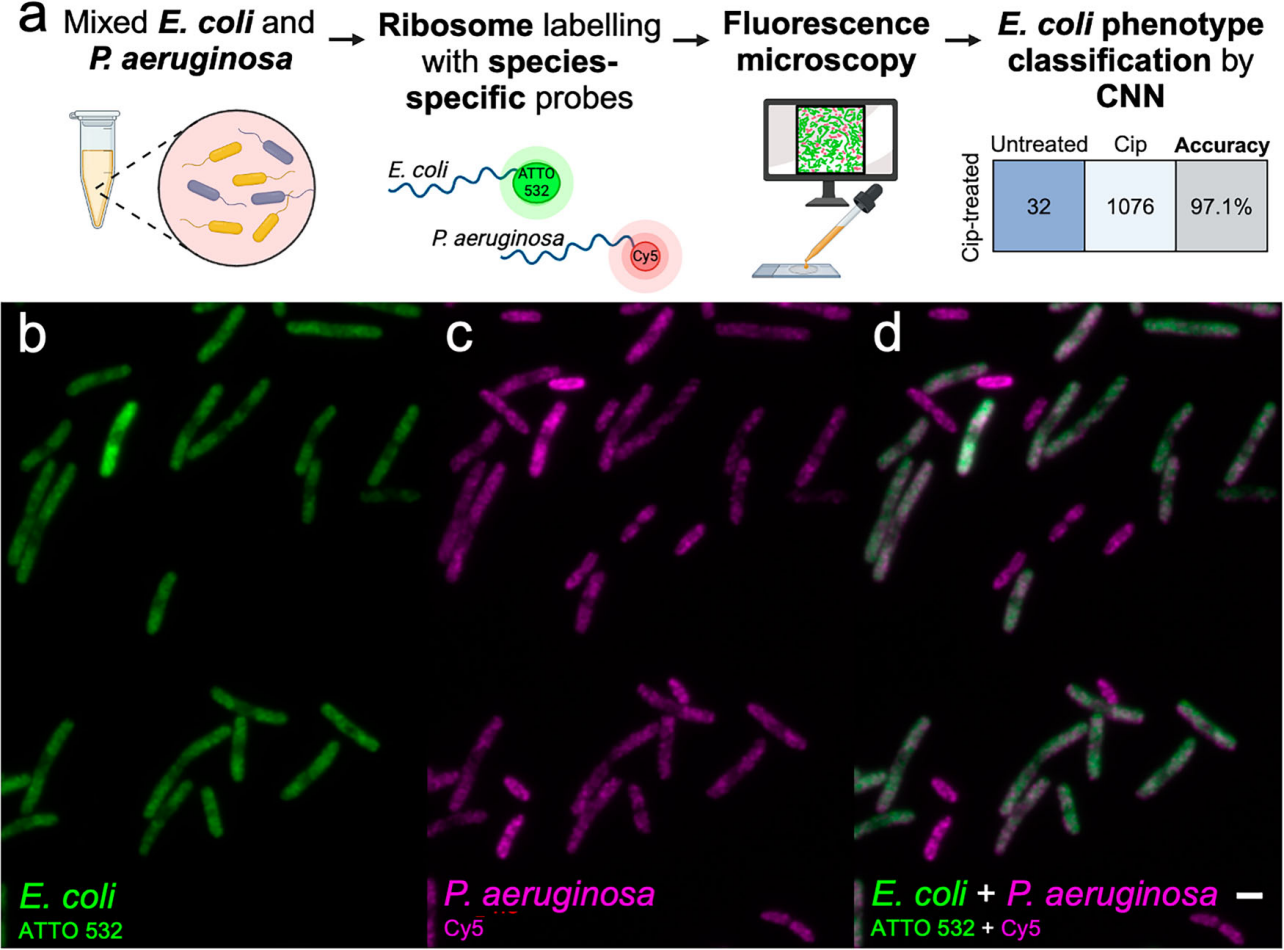
Species-specific probes can be used for simultaneous species and ribosome phenotype phenotype identification. **a** A one-to-one mixed sample of fixed and permeabilised *E. coli* and *P. aeruginosa* cells was mixed and suspended in hybridisation buffer with species-specific FISH probes targeting the 16S rRNA. The *E*. coli cells had been treated with 1 × EUCAST ciprofloxacin for 30 min to develop a ciprofloxacin-treated ribosome phenotype. The *E. coli*-specific probe was labelled with an ATTO-532 fluorophore while the *P. aeruginosa-*specific probe was labelled with a Cy5 fluorophore. After the sample was imaged on a fluorescence microscope, the *E. coli* cells were segmented and passed to the ciprofloxacin phenotype classifier, which identified them as ciprofloxacin-treated with 97.1% accuracy. **b** The *E. coli* cells can be visualised with the ATTO-532 fluorophore (green), illuminated with 532 nm excitation. **c** The *P. aeruginosa* cells can be visualised with the Cy5 fluorophore (magenta), illuminated with 647 nm excitation. There is some signal in the *E. coli* cells, at approximately half the brightness of the *P. aeruginosa* cells. **d** The composite image showing both ATTO-532 (green) and Cy5 (magenta) channels. The scale bar (white) is 2 μm.

Compared to conventional fluorescent stains or bright-field imaging, one advantage of the ribosome phenotype method is the potential to use species-specific FISH probes to simultaneously visualise the ribosome distribution and identify the bacterial species. We chose to demonstrate this with *Pseudomonas aeruginosa* and *Escherichia coli*, both Gram negative bacilli and 2 of the 6 leading pathogens associated with AMR deaths^3^. To test this capability, we moved from using the EUB338 probe, which targets a sequence conserved in all eubacteria, to FISH probes designed to target species-specific regions of the 16S ribosomal RNA in *E. coli*^42^ and *P. aeruginosa*^43^. A one-to-one mixture of fixed and permeabilised *E. coli* and *P. aeruginosa* cells was incubated in a hybridisation buffer with an equal concentration of each species-specific probe (Fig. 6a). The *E. coli* probe was labelled with ATTO-532 and the *P. aeruginosa* probe with Cy5. The *E. coli* sample was treated with 1 × EUCAST ciprofloxacin for 30 min to develop a ciprofloxacin-treated phenotype whereas the *P. aeruginosa* sample had not been exposed to antibiotics.

The *E. coli* ATTO-532 signal was highly specific for the elongated, ciprofloxacin-treated *E. coli* cells (Fig. 6b). The characteristic ciprofloxacin phenotype from nucleoid compaction can be visualised. The *P. aeruginosa* Cy5 signal can be seen most clearly in the *Pseudomonas* cells, although the probe can also be seen in the *E. coli* cells, at about half the fluorescence intensity (Fig. 6c). Overall, the species-specific probes can be used to differentiate *E. coli* and *P. aeruginosa* in a mixed sample (Fig. 6d).

When the *E. coli* cells imaged in this experiment were segmented from the ATTO-532 image and put through the MG1655 1 × EUCAST ciprofloxacin phenotype classifier, the CNN identified them as ciprofloxacin-treated with 97.1% balanced accuracy (1076 of 1108 cells). Therefore, ribosome phenotyping can be used with FISH probes targeting species-specific sequences to identify species and antibiotic phenotype from the same image dataset.

## Discussion

We have shown that ribosome-targeted FISH probes can be used to visualise intracellular antibiotic response phenotypes in *E. coli* that differ based on the mechanism of action of the antibiotic, and that these phenotypes can provide single-cell AST data with a single label. We demonstrated that distinct ribosome antibiotic response phenotypes exist for four antibiotics from clinically relevant classes, and that these can be learned by a CNN with >95% accuracy. In clinical isolates of *E. coli*, we found that the antibiotic response can be more complicated, and it cannot be assumed that the resistant strain will always resemble the untreated phenotype. However, by using a model trained to identify the phenotypes of clinical isolates, we achieved an average single-cell classification accuracy of 94.9%. The ribosome phenotype classification method was also shown to extend to clinical strains not shown to the model in the training data and treated at a different antibiotic concentration. If deployed in a real-world AST, as the CNN models have access to training data from additional susceptible and resistant strains, the performance of the ribosome phenotype classifier would only be expected to improve.

In the context of a diagnostic test, our current single-cell accuracy means that with only 2 cells, we can differentiate a susceptible from a resistant sample with 99% confidence and can identify mixed infections with a sample of 12 bacterial cells. On a realistic scale of between 10 to 100 bacteria captured from a dilute sample such as blood or cerebrospinal fluid, this level of accuracy could enable confident diagnosis even in less ideal imaging conditions. Together with the previous deep-learning-based AST^30^ based on nucleoid and cell membrane staining, our ribosomal method achieves similar accuracy while requiring only a single fluorescent label. Our results using the ribosome phenotyping method provide additional validation for the use of CNNs to detect single-cell changes associated with antibiotic susceptibility.

The previous deep-learning-based AST also showed that it could provide equivalent information to growth-based assays^30^ through the relationship between the proportion of cells classified as antibiotic-susceptible and the MIC of the strain. Here, using the ribosome phenotype, we also find a strong relationship between the MIC of the clinical isolate and its morphology after antibiotic treatment with ciprofloxacin. While the previous method used untreated lab-strain *E. coli* as a proxy for the resistant phenotype, we found that our MG1655-trained ribosome phenotype classifier had low accuracy when classifying resistant strains with a diffuse nucleoid but elongated cell shape. This could be because the MG1655 treated with ciprofloxacin is longer than untreated cells, and the ribosome phenotype classifier is classifying predominantly by cell length. Given the difference we found between clinical-strain and lab-strain phenotypes, we recommend that antibiotic response phenotypes are characterised in clinical isolates when possible, although lab strains can be used as a starting point. Our results applying models to clinical isolates treated at different concentrations than the training data show some transferability, but sufficiently similar training data is required to have high accuracy.

The antibiotic response phenotypes of pathogenic *E. coli* are diverse, and there are many insights that could be learned from the 47,704 high-resolution single-cell images that were obtained for this study and are being made available for research (see *Data Availability section*). Generating these large, curated datasets of high-resolution bacterial images is time-intensive, because many high-throughput systems are optimised for eukaryotic cells, but they can be powerful in developing our understanding of bacterial antibiotic response. Many mechanisms contribute to ciprofloxacin resistance beyond mutations in target genes, such as the expression of efflux pumps and plasmid-borne *Qnr* genes^11,41,44^ and the 7 clinical isolates we tested represent a small fraction of the diversity present in pathogenic *Escherichia coli*. Beyond the heterogeneity in response within a single sample, we found that two isolates with the same MIC (0.25 mg/L) but different genotypes showed different morphologies and were classified as resistant at different rates (6% vs. 18%). These divergent responses to antibiotic treatment are an area of active research and are important for reliable phenotypic detection of AMR^45^.

Compared to growth-based assays^15,16,18^, this method is limited to assessing the ribosome phenotype at a single timepoint because of the fixation and permeabilization steps necessary for FISH. Compared to genotypic ASTs, a challenge of the ribosome phenotyping method is that it requires separate interrogations for each antibiotic. This could be parallelised but introduces complexity to the testing procedure. Using an imaging-based phenotyping method also requires a microscopy platform and a method for sample preparation. However, this method might be especially valuable in deep learning-assisted drug discovery, and in studies determining the mechanism of action of antibiotics^46^. The advantages of the ribosome-labelling FISH approach are the plethora of structural features that are amenable to deep-learning-based classification and the potential for simultaneous species ID with species-specific FISH probe sets, which has been demonstrated for many species and contexts^47–50^. We have shown that species-specific probes can be used to differentiate *E. coli* from *P. aeruginosa* while simultaneously classifying the ribosome phenotype. The combination of live-cell growth rate and fixed-cell phenotypic data could be even more powerful than what we have shown in assessing a cell’s antibiotic response.

Here, we demonstrate the accuracy of a ribosome phenotype classifier on *E. coli* clinical isolates treated with ciprofloxacin. To have greater clinical utility, this method will need to be extended to other bacterial species and antibiotics. FISH probes targeting the ribosomal RNA have been used to identify a variety of Gram positive and negative species for clinical applications^47,48,51^. In the future, these probes could be combined with ribosome phenotyping for many of these species. It is likely that the extension of this method from *E. coli* to other Gram-negative bacilli will be more straightforward, while the smaller size of cocci and lower permeability of Gram-positive bacteria to FISH probes^52,53^ may be a greater challenge. Similarly, we have shown that three antibiotics with intracellular targets (ciprofloxacin, gentamicin, chloramphenicol) and one antibiotic targeting the cell wall (carbenicillin) cause characteristic ribosome phenotypes that can be identified by a CNN. We expect that this method can be extended to other antibiotics, so long as they reliably induce a visible change in the ribosome phenotype within the time scale of the test. As was shown for gentamicin (Fig. S1) and carbenicillin (Fig. S2), the benchmark treatment concentration may differ for each antibiotic. The length of antibiotic treatment may also need to be adjusted, especially for slow-growing species. Despite these challenges, when used in combination with bacterial genotyping, a single-cell imaging assay like this one could also be used to profile new resistance-associated mutations. This work serves as a guide for how deep learning can be used with fluorescence microscopy to learn intracellular phenotypes with high levels of accuracy, which can be applied to different species, and antibiotics, as well as to many biological, clinical and biotechnological applications.

In the context of ultra-rapid ASTs, although cytological profiling with classical statistics or machine learning has advantages in interpretability^18,29^, the expected diversity of ribosome phenotypes in response to antibiotic treatment in different bacterial strains and species is one of the motivators for a CNN-based phenotypic AST, because a CNN can be expected to improve in performance when it is able to learn from additional, real-world data^54^.

By combining our single-cell ribosome-based assay with highly efficient microfluidic capture chips^50^, an AST could be performed on cells captured directly from the clinical specimen, eliminating the need for lengthy culture steps, and could use multiplex FISH probes that bind to species-specific regions on the ribosomal RNA to report both the species ID and antibiotic susceptibility data.

## Methods

### Bacterial strains and sample preparation

*Escherichia coli* MG1655, a lab-adapted non-pathogenic K-12 derivative, was used as the reference strain for characterising antibiotic-susceptible ribosome phenotypes. Clinical strains were grown from stored blood culture isolates obtained for diagnostic and research purposes by the Microbiology Laboratory of the Oxford University Hospitals NHS Foundation Trust, Oxford, UK. All clinical isolates had been sequenced on the Illumina platform and AMR genotypes were assigned using the ResFinder^55^ database with Abricate v0.9.8^56^ (--min-id 95 –min-cov 95) as part of a previous study^57^ (Table 1). A biological replicate was defined as a culture grown from an individual colony on an agar plate.

The minimum inhibitory concentration (MIC) of each strain was tested in biological duplicate according to the broth microdilution method^58^ (Table 1, Table S1). The MIC was defined as the lowest antibiotic concentration inhibiting growth when the cultures were incubated overnight in Mueller-Hinton broth at 37 °C.

Bacterial cultures were prepared in a shaking incubator at 37 °C in 5 mL lysogeny broth (MG1655) or Mueller-Hinton broth (clinical isolates, as is standard in clinical microbiology labs) until reaching logarithmic growth, or OD_600nm_ of 0.2. Then, antibiotics were added to reach the specified concentration (see EUCAST Breakout Points, Table S1) and the samples were returned to the incubator for the 30-minute treatment period. Samples were then fixed in 2% paraformaldehyde for 20 min. After fixation, the samples were centrifuged (3 min) and the cell pellets were washed once with PBS, then re-centrifuged (3 min) and re-suspended in 5 mL PBS before being split into 1 mL aliquots and permeabilised in 500 µL absolute ethanol (20 min) before being stored at−20 °C until use.

Before imaging, the cells were centrifuged (3 min) to remove the ethanol supernatant, washed with 500 µL PBS, and resuspended in hybridisation buffer (20% v/v formamide, 0.9 M NaCl, 20 mM Tris pH 7.5, 0.01% SDS w/v). For labelling, 4’,6-diamidino-2-phenylindole (DAPI, 1 µg/mL) and 25 nM EUB338-Cyanine3 were added to the solution and the sample was incubated for 20 min at room temperature. The ssDNA EUB338-Cyanine3 FISH probe has the sequence Cyanine3 – 5’ – gct gcc tcc cgt agg agt – 3’ (Sigma Aldrich). Following incubation, the samples were washed with 500 µL PBS and resuspended in 150 µL PBS. This FISH procedure results in robust cell labelling (Fig. S13). From the start of antibiotic treatment to the start of imaging, the protocol takes approximately 2 h.

For the species identification experiments, we used probe sequences that had been used for the identification of *E. coli*^42^ and *P. aeruginosa*^43^. The *E. coli* probe has the sequence ATTO-532 – 5’ – gca aag gta tta act tta ctc cc – 3’ (Sigma Aldrich). The *P. aeruginosa* probe has the sequence Cy5 – 5’ – gga cgt tat ccc cca cta t – 3’ (Sigma Aldrich). These probes were incubated with the sample at a higher concentration of 2 μM as was used in previous work^49^.

### Image acquisition

Samples were imaged on agarose pads prepared with 1.5% (w/v) high-purity agarose (Bio-Rad, catalogue number 1613101) in distilled water. Images were collected on the Nanoimager-S microscope (ONI, Oxford, UK) with a 100 × oil-immersion objective in multi-acquisition mode. The DAPI stain was illuminated by a 405 nm laser in epifluorescence mode at a laser power of 5.1 kW/cm^2^ for an acquisition time of 20 ms. The Cyanine 3 fluorophore was illuminated with a 532 nm laser in epifluorescence mode at a laser power of 16.5 kW/cm^2^ for 20 ms. For the species-specific probes, image quality was improved with an acquisition time of 100 ms. The ATTO-532 fluorophore was illuminated with a 532 nm laser in epifluorescence mode at a laser power of 16.5 kW/cm^2^ for 100 ms and the Cyanine 5 fluorophore was illuminated with a 647 nm laser in epifluorescence mode at a laser power of 5 kW/cm^2^ for 100 ms. For each replicate, we collected as many high-quality fields of view as was possible. No calculation was carried out to pre-select the sample size.

### Image processing and segmentation

Each field of view was segmented using Napari-BacSeg^37^, a user-friendly bacterial analysis platform that allows microscopy images to be segmented using machine learning models, such as CellPose^36^ and OmniPose^59^. BacSeg can also be used to train custom CellPose or OmniPose models to improve segmentation performance and minimise the need to curate segmentations or fix segmentation errors. Within the software, the resulting segmentations can be easily curated and then exported in multiple formats to facilitate downstream analysis. Descriptive statistics of the segmented bacteria can also be computed and exported. The BacSeg Napari^60^ plugin can be installed from from the Napari Hub, the Python package manager PyPi, or GitHub (https://github.com/piedrro/napari-bacseg).

For our segmentations, custom CellPose^36^ models were trained on our 532 nm ribosome data for 100 epochs using the standard Napari-BacSeg hyperparameters to improve segmentation performance; these were then used for cell segmentation. Cells on the edge of the image, overlapping cells, vertical cells, or cells outside of the focal plane were removed from the final dataset during the segmentation and curation process.

### Cell phenotypes

From the curated segmentations, cell lengths, widths, and midlines were generated using the ColiCoords^61^ plugin within Napari-BacSeg^37^ with 10 midline vertices. For each cell, the intensity in each channel is normalised from 0 to 1 and the mean intensity is calculated for each channel for 100 bins along this midline. For a population of cells, the mean intensity and the standard deviation of the intensity is calculated for each of these 100 bins. This provides a mean intensity for each bin along the long axis of the cell. Because the cell lengths cannot be assumed to be normally distributed, hypothesis testing was conducted with the Mann-Whitney non-parametric test with a significance value of 0.05 in Origin Pro 2021.

### Neural network training

Images and segmentation maps were exported from Napari-BacSeg^37^ to create standardised 64 × 64 pixel images of each cell zero-filled outside the segmentation boundary in order to be passed to the CNN. For cells longer than this size, we tested trained on cropped cells and models trained on cells that were resized while maintaining the aspect ratio if the bounding box length or width was greater than 64 pixels (Fig. S5). To preserve the intracellular definition, models presented in the main *Results* are for models without resizing. To account for different staining and illumination brightness, histogram normalisation was applied to each image. The images were randomly rotated, flipped, and translated with geometric and noise transforms from the Albumentations package^62^ before being loaded into the neural network (Fig. S4).

The convolutional neural network was built with PyTorch^63^. Each model was run for 100 epochs with the batch size and learning rate optimised by Optuna^64^ for the each dataset. The training datasets were split 0.80/0.20 into training and validation datasets. The validation and testing datasets were balanced by class to reduce classification bias. Because the task was a single-label binary classification, EfficientNetB0^65^ with the Cross-Entropy loss function was used as the neural network structure. The Adam function^66^ was used for adaptive learning rates.

All accuracies reported are from holdout datasets, meaning that the model was independently trained and validated on data from several biological replicates and tested on images from a previously unseen biological replicate.

### Accuracy metrics and sample size simulation

Plots and phenotype statistics such as the Mann–Whitney non-parametric test were done using Origin Pro 2021. The Mann–Whitney non-parametric test was chosen because the phenotype measurements cannot be assumed to be normally distributed, as they are composed of cells at different stages of the cell cycle. The cell classification simulation to determine sample size was calculated using MATLAB R2022b.

*1. Balanced Accuracy*. All accuracies are reported with the 95% confidence interval, ± 2σ. Here we define resistant cells as “Positives” and susceptible cells as “Negatives.” For a one-class binary classification task,

$${Accuracy}=\frac{{TruePositives}}{{TruePositives}+{FalseNegatives}}$$

For a two-class classification task, given that:

$${Sensitivity}=\frac{{TruePositives}}{{TruePositives}+{FalseNegatives}}$$

and

$${Specificity}=\frac{{TrueNegatives}}{{TrueNegatives}+{FalsePositives}}$$

then

$${BalancedAccuracy}=\frac{1}{2}({Sensitivity}+{Specificity}).$$

$${PositivePredictiveValue}=\frac{{TruePositives}}{{NumberofPositiveCalls}}$$

$${NegativePredictiveValue}=\frac{{TrueNegatives}}{{NumberofNegativeCalls}}$$

*2. Sample Size Simulation*. Given a sample of cells of size *N* and a certain proportion of susceptible and resistant cells (100:0, 50:50, 0:100), we simulated the measured Resistant Fraction and 95% confidence interval. We simulated random samples of 10,000 susceptible and resistant cells at the defined proportions and transformed them into detected samples using the accuracy of our susceptible-resistant classifier. A resistant cell was detected as resistant 98.8% of the time (sensitivity) and a susceptible cell was detected as susceptible 91.7% of the time (specificity). Random populations of between 1 and 40 cells were sampled and the Resistant Fraction (resistant cells/total cells) was calculated. After 1000 trials, the mean Resistant Fraction and 99% confidence interval (2.58σ) was plotted for each sample size. We defined the minimum sample size as the smallest sample for which the 99% confidence intervals did not overlap.

### Ethics

Ethical approval for the use of clinical isolates processed by the John Radcliffe Hospital microbiology laboratory in the development of diagnostic assays was granted by the UK’s Health Research Authority (London – Queen Square Research Ethics Committee [REC reference^17^:/LO/1420]).

## Supplementary information

Supplemental Material

## Data Availability

Cell images, metadata, and source data for all figures are available at: https://zenodo.org/records/11656505. All other data are available from the corresponding author on reasonable request.

## Code availability

Code for model training is available at: https://github.com/KapanidisLab/ribosome_phenotype_classification^67^.
