# Supplementary material for: Ribosome phenotypes for rapid classification of antibiotic-susceptible and resistant strains of Escherichia coli
Source: Commun Biol. 2025 Feb 26;8:319. doi: 10.1038/s42003-025-07740-6 (PMC11865533; doi:10.1038/s42003-025-07740-6)
Supplement: Supplementary file 1 — Supplemental Material [file 42003_2025_7740_MOESM1_ESM.pdf]

Supplementary Material

Ribosome phenotypes for rapid classification of antibiotic-susceptible and resistant strains of *Escherichia coli*

Alison Farrar, Piers Turner, Hafez El Sayyed, Conor Feehily, Stelios Chatzimichail, Sammi Ta, Derrick Crook, Monique Andersson, Sarah Oakley, Lucinda Barrett, Christoffer Nellåker, Nicole Stoesser, and Achillefs Kapanidis

	Antibiotic Minimum Inhibitory Concentration (mg/L)			
	**Chloramphenicol**	**Gentamicin**	**Ciprofloxacin**	**Carbenicillin***
MG1655<br>K-12 Lab Strain	3.75	0.125	0.016	20
**EUCAST Breakout Point**	**>8**	**>2**	**>0.5**	**>8**

Table S1. MG1655 MICs and EUCAST breakout points for chloramphenicol, gentamicin, and ciprofloxacin. The minimum inhibitory concentration (mg/L) for *E. coli* MG1655 is shown for chloramphenicol, gentamicin, ciprofloxacin, and carbenicillin. These concentrations were used to determine antibiotic treatment concentrations, e.g. 1× EUCAST = 8 mg/L chloramphenicol, 1× EUCAST = 0.5 mg/L ciprofloxacin, 20× EUCAST = 40 mg/L gentamicin, and 3× EUCAST = 24 mg/L carbenicillin. MICs were determined by broth microdilution (see *Methods: Bacterial strains and sample preparation*). NB*: Carbenicillin is a β-lactam without a EUCAST breakpoint. Its mechanism of action is closely related to ampicillin and other penicillins, with the main benefit of increased stability, but higher cost. Here we reference the EUCAST breakout point for penicillins without β-lactamase inhibitors.

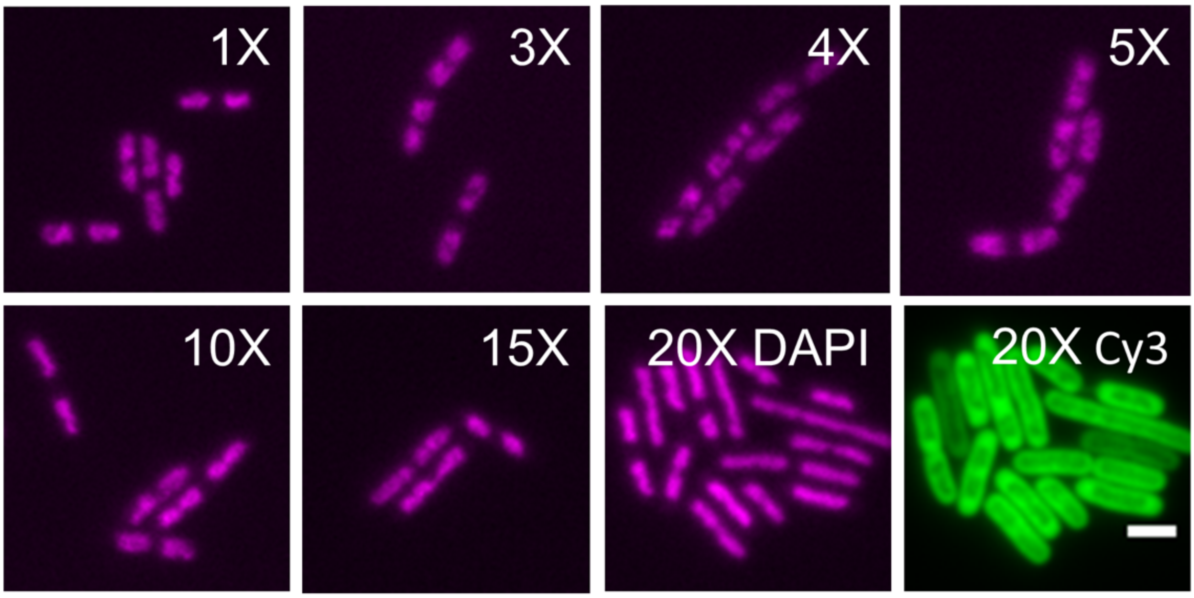

Figure S1. Phenotypic changes associated with gentamicin treatment for 30 minutes at various concentrations from 1× to 20× EUCAST. Representative images of *E. coli* MG1655 treated with gentamicin in LB media for 30 minutes, labelled with the gentamicin concentration in multiples of the EUCAST breakpoint (see Table S1). Images show the nucleoid (DAPI) unless otherwise labelled. Samples were labelled with DAPI and EUB338-Cy3 as described in the *Methods*. Scale bar, 2 µm.

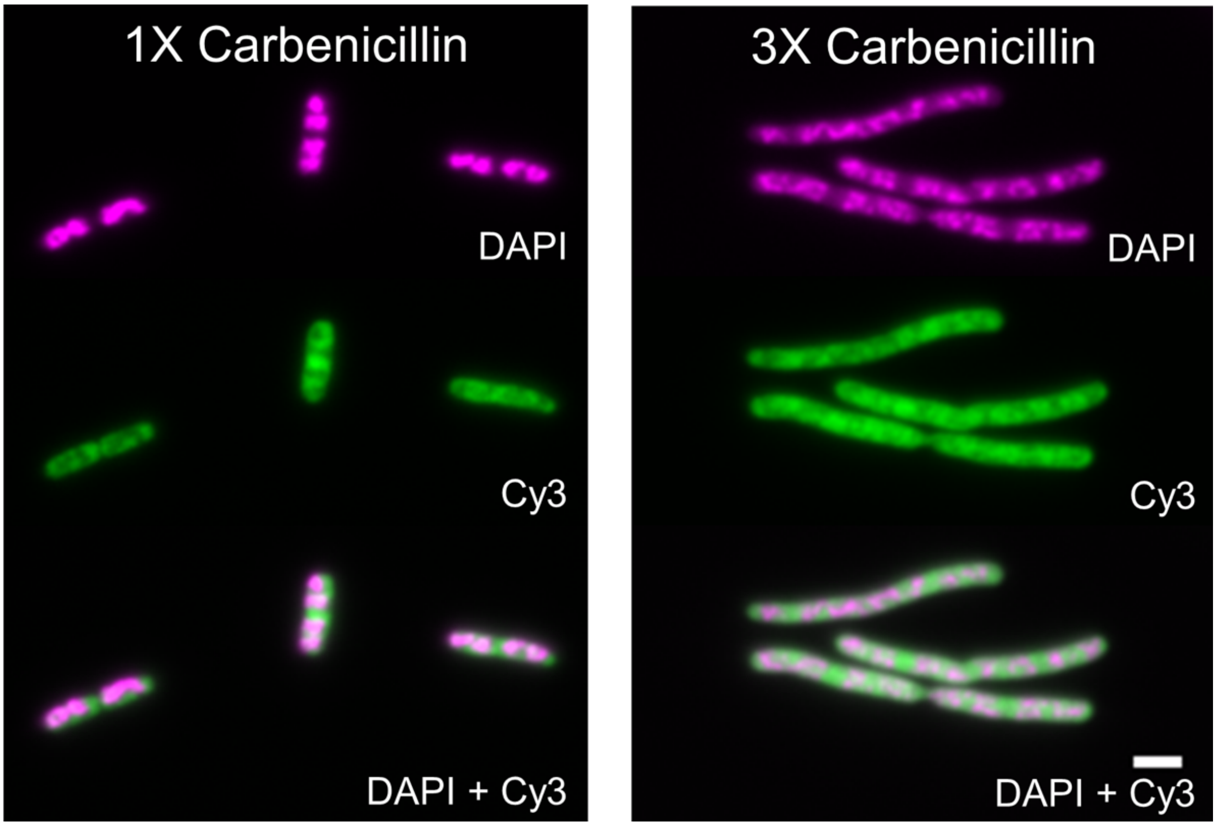

Figure S2: Phenotypic changes associated with carbenicillin treatment for 30 minutes at 1× and 3× EUCAST. Representative images of *E. coli* MG1655 treated with carbenicillin in LB media for 30 minutes, labelled with carbenicillin concentration in multiples of the EUCAST breakpoint (see Table S1). Images show the nucleoid (magenta, DNA stained with DAPI); the ribosome phenotype (green, ribosomes labelled with EUB338-Cy3 probes); and the combined DNA and ribosome signal. Scale bar, 2 μm.

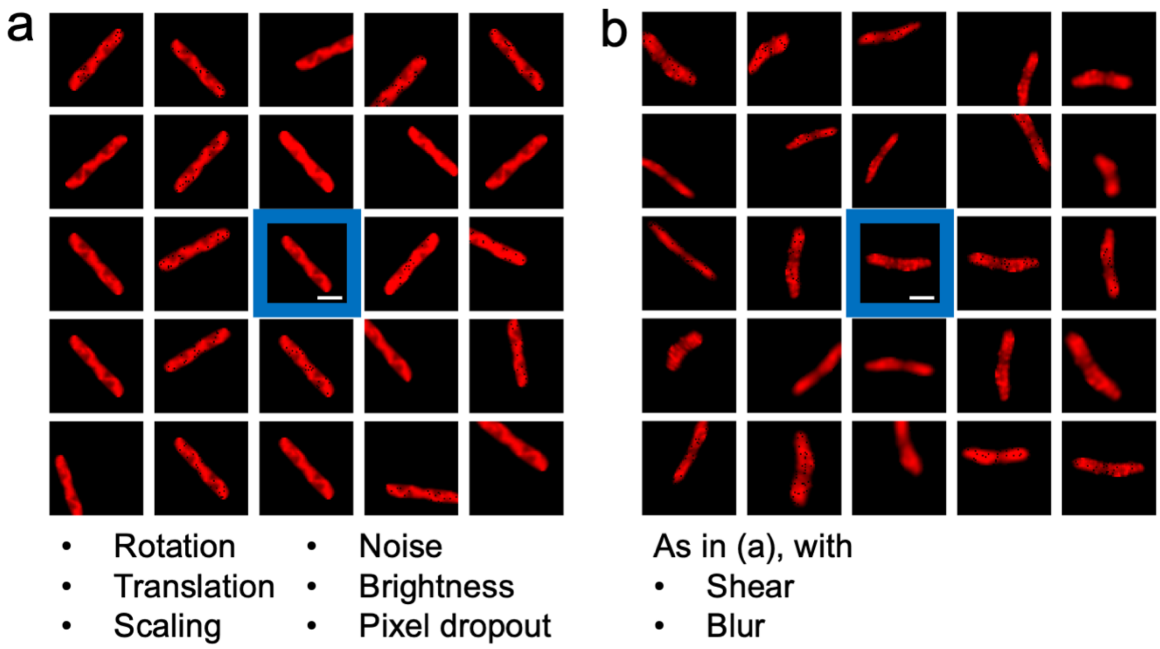

Figure S3. Image augmentations with and without shearing and blurring. Raw images (centre, highlighted in blue) and a selection of resulting augmented images are shown for representative cells. Scale bar, 2 µm.

(a) Some of the augmentations can be seen, such as random rotations, translations, scaling, Gaussian noise, brightness adjustments, and pixel dropout.

(b) As in (a), with additional random shear and blur.

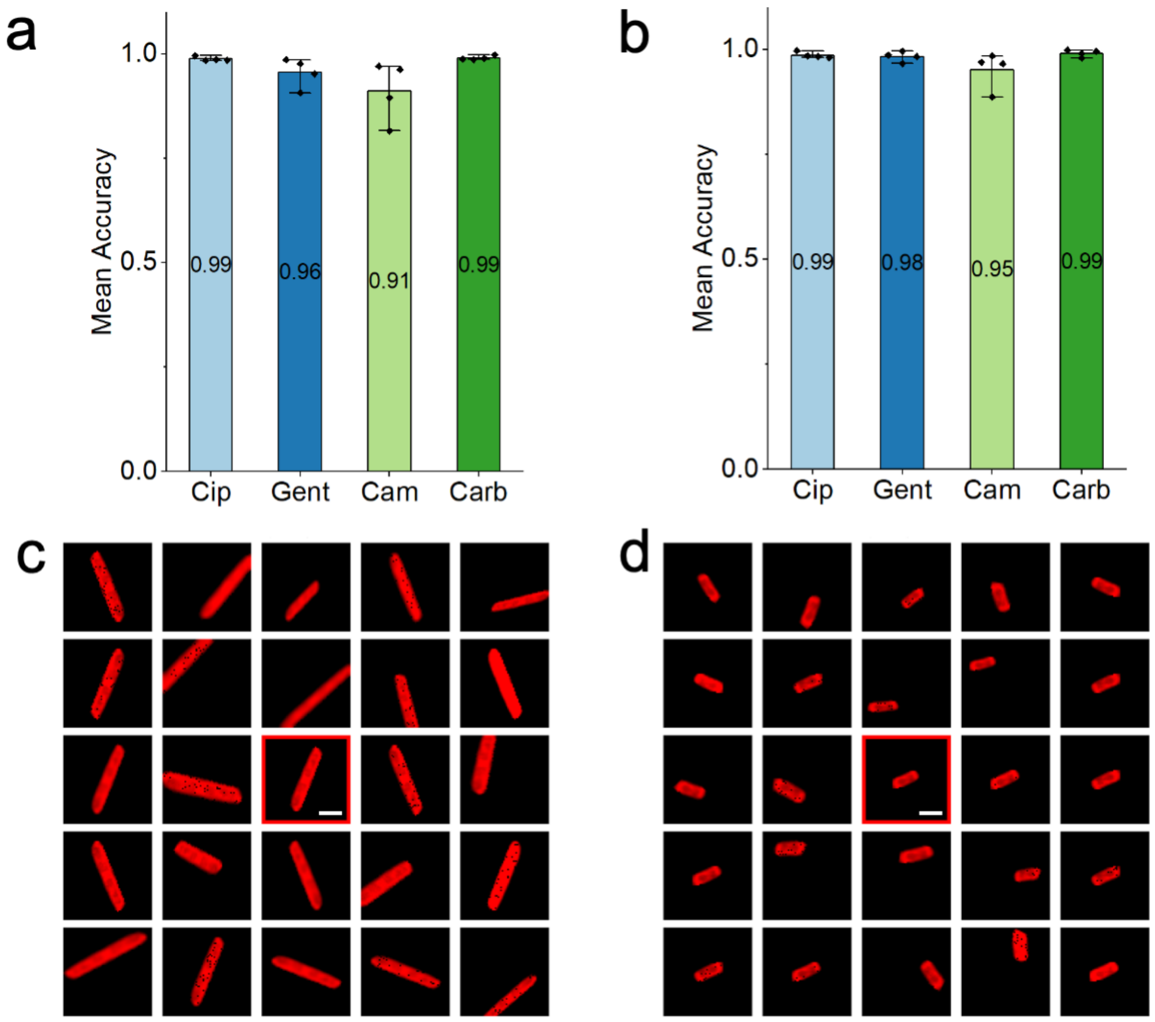

Figure S4: Gentamicin and chloramphenicol model training is hindered by shearing and blurring augmentations.

(a) The balanced accuracy of the ribosome phenotype classifier is shown for each antibiotic using the augmentations demonstrated in Figure S3(b), which include shear and blur. Each point represents a biological replicate. The mean balanced accuracy is shown as on each column and the error bars indicate the 95% confidence interval of the mean on the four biological replicates. The mean balanced accuracy for gentamicin is 0.96 and the mean balanced accuracy for chloramphenicol is 0.91.

(b) As in (a), for a model trained with the augmentations demonstrated in Figure S3(a), which do not include shear or blur. The mean balanced accuracy for gentamicin has increased to 0.98 and the mean balanced accuracy for chloramphenicol has increased to 0.95.

(c) Representative augmentations with shear and blur for a gentamicin-treated cell (raw image highlighted in red box, centre). The ribosome phenotype is obscured in several of the augmented images. Scale bar, 2 μm.

(d) Representative augmentations without shear and blur for a chloramphenicol-treated cell (raw image highlighted in red box, centre). The ribosome phenotype can be seen in most augmented images, despite added noise and brightness adjustments. Scale bar, 2 µm.

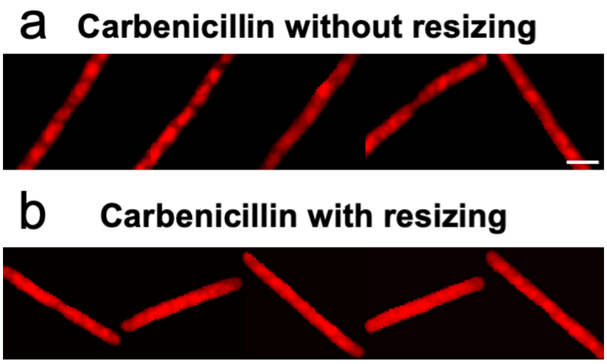

Figure S5: Resizing obscures the ribosome phenotype in extremely long cells.

(a) Representative images of carbenicillin-treated cells without resizing. Scale bar, 2 µm. Before the image is passed to the CNN, the segmented cell is centred, but the cell poles are cropped by the standardized size. However, the regions of decreased ribosome density (nucleoid regions) can be seen throughout the cell.

(b) Representative images of carbenicillin-treated cells with resizing. These segmented cells are resized with preserved aspect ratio to fit in 64×64 images before being passed to the CNN. As a result, the intracellular definition and scale is lost for the longest cells.

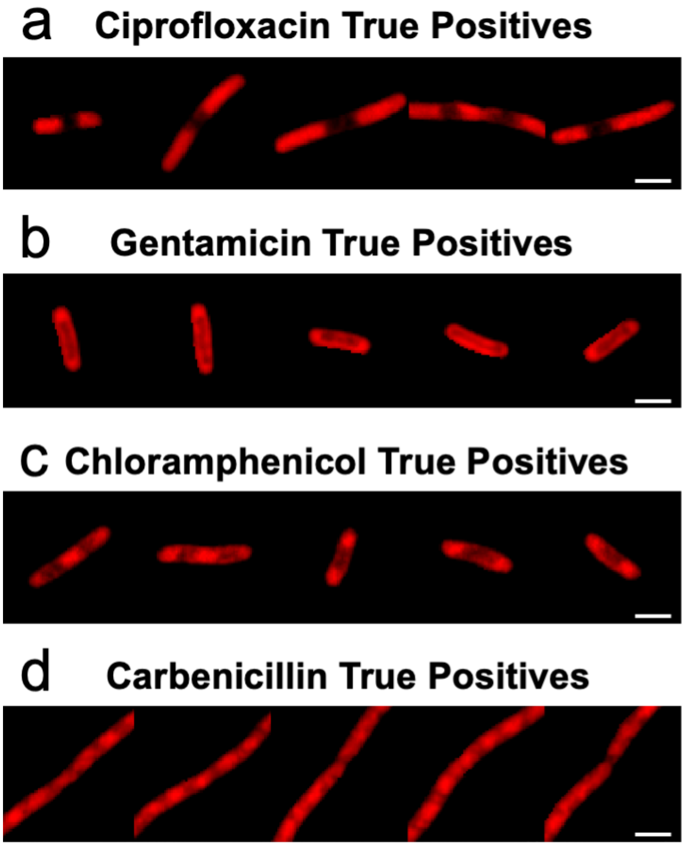

Figure S6. Representative classifications for the ciprofloxacin, gentamicin, and chloramphenicol phenotype models. Example ribosome phenotypes are shown for correctly classified *E. coli* MG1655 cells treated with ciprofloxacin (a), gentamicin (b), chloramphenicol (c), and carbenicillin (d) as described in the *Methods*. Scale bar, 2 µm. The ciprofloxacin cells show elongated cells with a central, compact nucleoid region. One image shows a cell where the poles have been cut off by the standardized 64×64 image passed to the CNN. The gentamicin cells show elongation and a nucleoid region that has compacted along the midline. The chloramphenicol cells show central, compact nucleoid regions. The carbenicillin cells show multiple nucleoid regions and filamentation. All of the carbenicillin-treated cells are longer than the 64×64 image passed to the CNN.

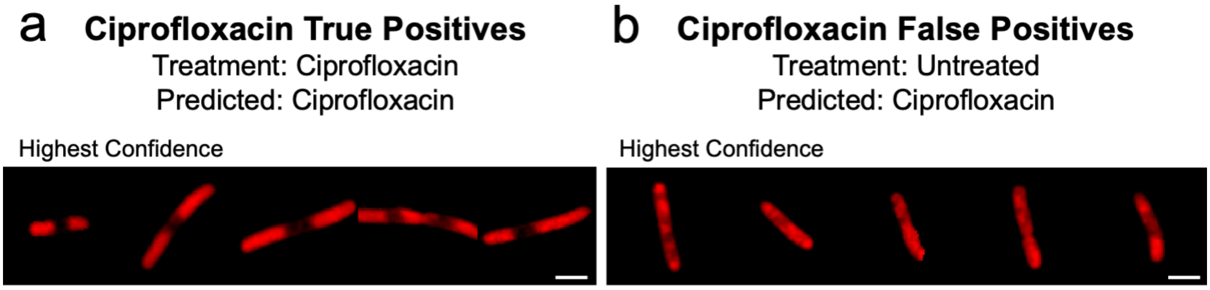

Figure S7. Images of cells classified as ciprofloxacin-treated by the model. Representative ribosome phenotypes of *E. coli* MG1655 are shown for (a) True Positives (ciprofloxacin-treated, predicted ciprofloxacin-treated) and (b) False Positives (untreated, predicted ciprofloxacin-treated) that were classified with the highest confidence by the model. Scale bar, 2 µm. The True Positive images show an elongated phenotype and a central nucleoid region with lower ribosome intensity, especially in the images classified with the highest confidence. The False Positive images that were misclassified are elongated, or have a central nucleoid region, or both. This suggests that the ciprofloxacin model is using cell length and the existence of a central nucleoid to classify cells as ciprofloxacin-treated.

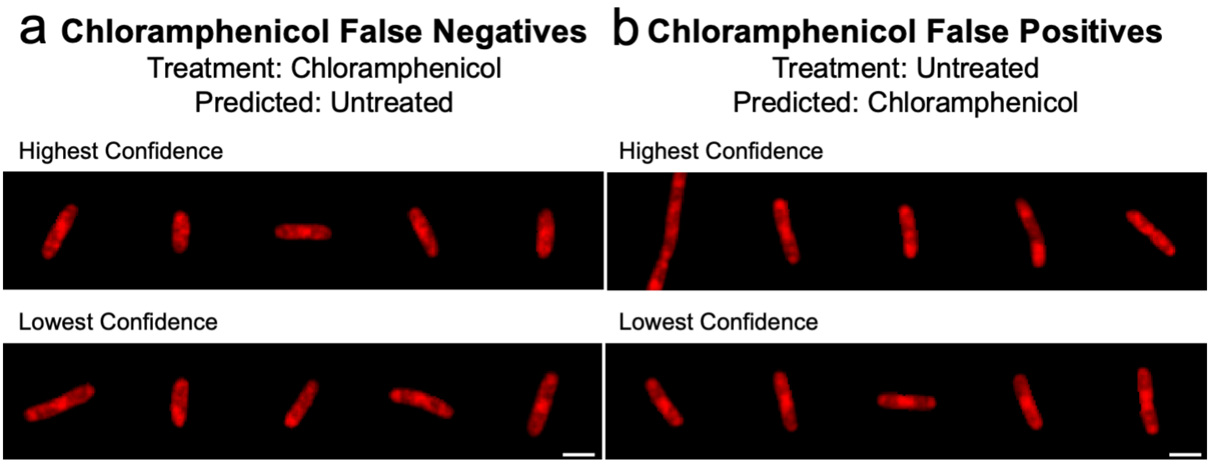

Figure S8. Images of *E. coli* MG1655 incorrectly classified by the chloramphenicol model. Example ribosome phenotypes of (a) False Negatives (chloramphenicol-treated, predicted untreated) and (b) False Positives (untreated, predicted chloramphenicol-treated). Scale bar, 2 μm. The False Negative cells show multiple, diffuse nucleoid regions in chloramphenicol-treated cells, whereas the False Positives show central nucleoid regions in untreated cells.

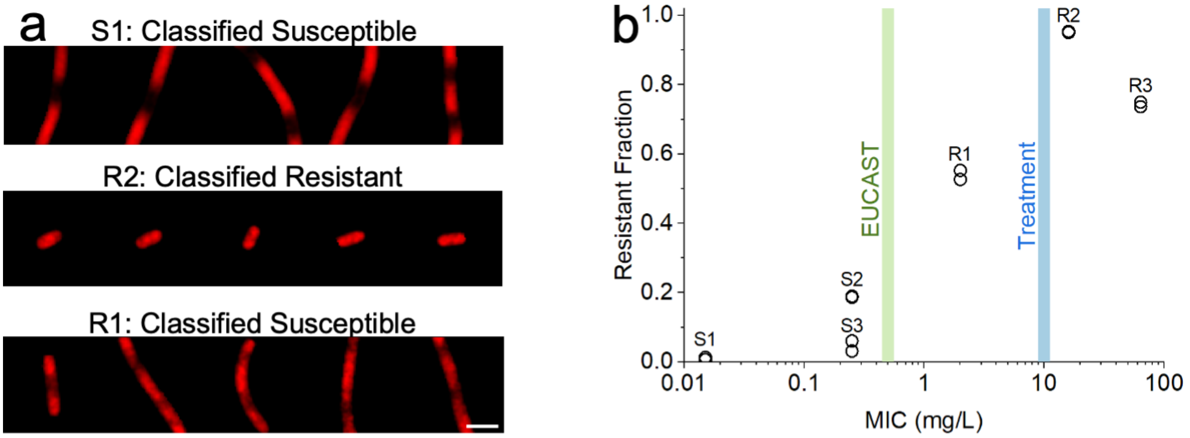

Figure S9. Classifier trained on *E. coli* MG1655 applied to clinical isolates has variable accuracy.

(a) Representative ribosome phenotypes from S1 cells correctly classified as susceptible, showing an elongated phenotype and compact nucleoid; R2 cells correctly classified as resistant, showing a phenotype more typical of untreated *E. coli* MG1655; and R1 cells incorrectly classified as susceptible, showing an elongated phenotype and diffuse nucleoid. Scale bar, 2 µm.

(b) The fraction of cells in the sample called Resistant by the MG1655 ciprofloxacin classifier is plotted against the MIC of the strain (mg/L) on a logarithmic scale. Each circle represents a biological replicate. The test dataset is composed of images from the six *E. coli* clinical isolates treated with 10 mg/L ciprofloxacin for 30 minutes. The EUCAST breakpoint (0.5 mg/L) and the treatment condition (10 mg/L) are shown with vertical lines. All strains with an MIC below the EUCAST breakpoint have a resistant fraction less than 0.2, whereas there is no clear relationship between the MIC and the resistant fraction for the resistant strains.

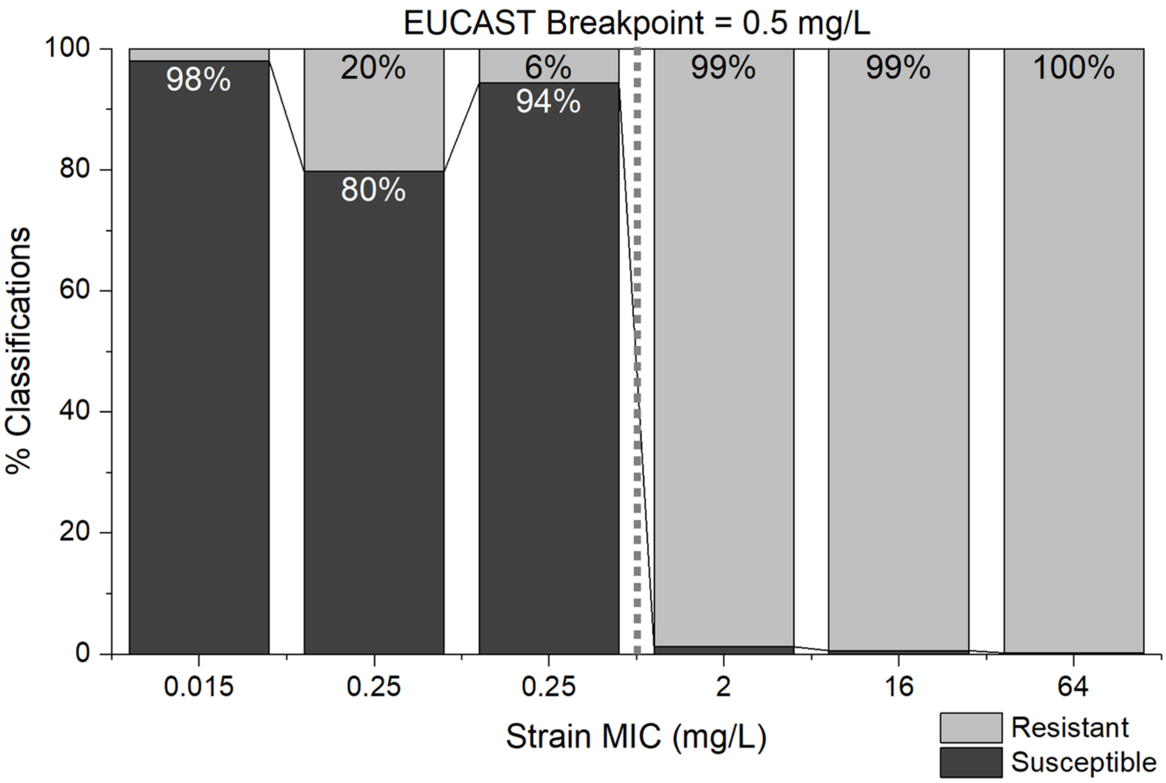

Figure S10. The sensitive-resistant model can differentiate susceptible (MIC < 0.5 mg/L) and resistant (MIC > 0.5 mg/L) clinical isolates. The percentage of cells in the test dataset classified as susceptible (dark grey) or resistant (light grey) is shown for each of the clinical isolates, ordered by the MIC of the strain. For strains with an MIC below the EUCAST breakpoint (0.5 mg/L), the % of susceptible classifications is greater than or equal to 80% (98%, 80%, 94%). For strains with an MIC above the EUCAST breakpoint, the % of resistant classifications approaches 100% (99%, 99%, ~100%).

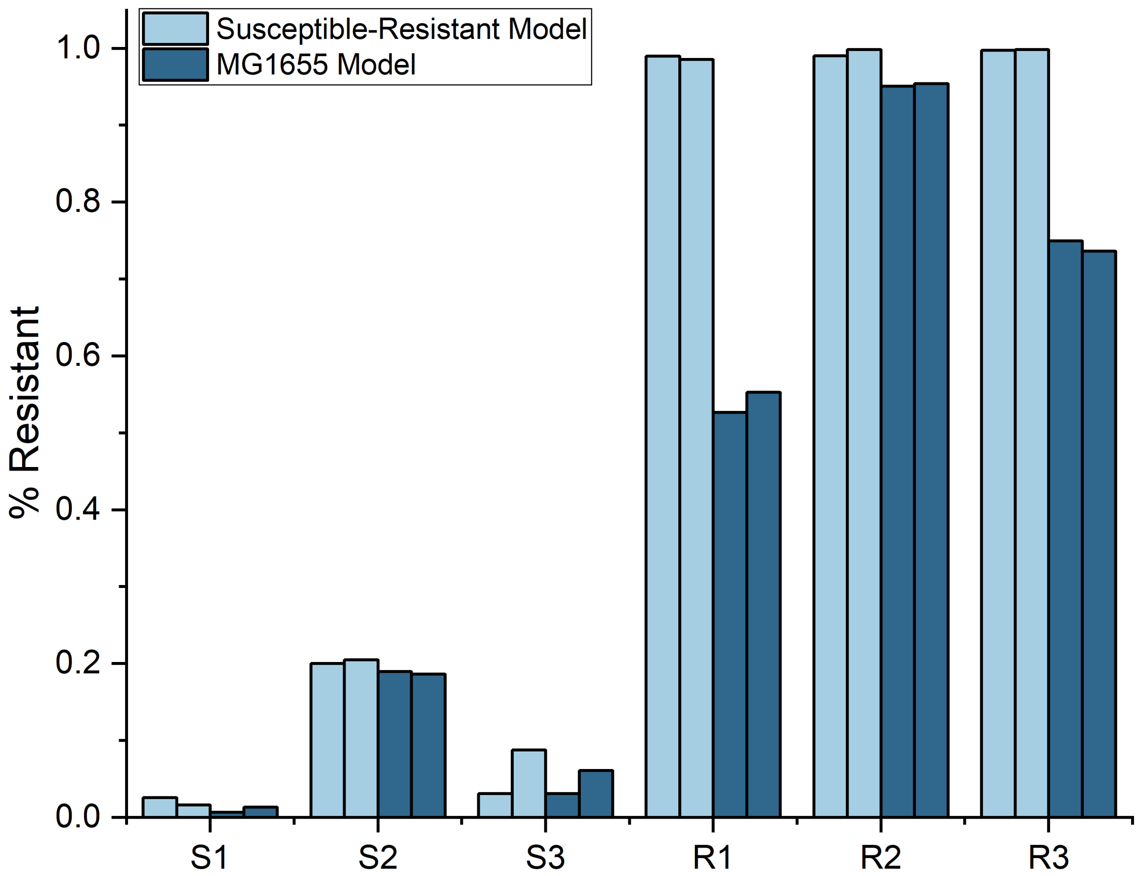

Figure S11. The susceptible-resistant model based on clinical isolates is more accurate than the MG1655 model. The fraction of cells classified as resistant (Resistant Fraction) is shown for each of the clinical strains (S1, S2, S3, R1, R2, R3), based on classifications by the susceptible-resistant model (light blue) or the MG1655 model (blue). Each bar represents a biological replicate. The test dataset is composed of images from the six *E. coli* clinical isolates treated with 10 mg/L ciprofloxacin (20× EUCAST) for 30 minutes. Compared to the MG1655 model, the susceptible-resistant model has similar or higher accuracy on all sensitive strains and is more accurate on all resistant strains.

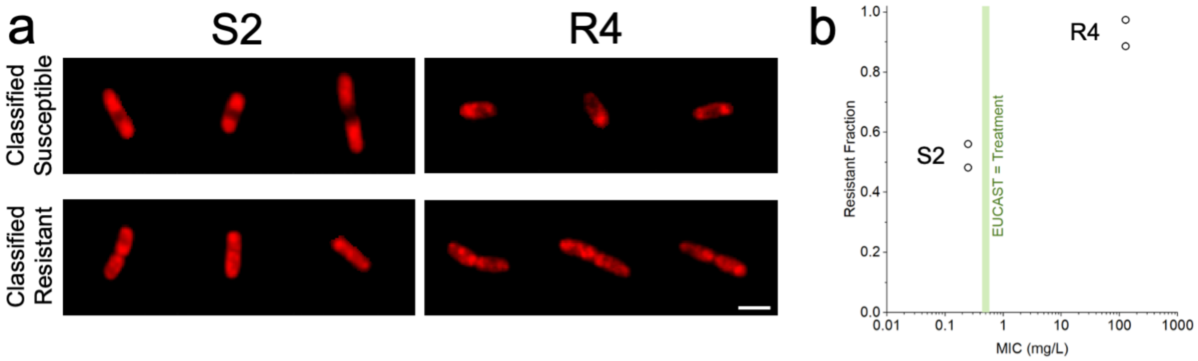

Figure S12. Classifier trained on clinical isolates treated at 20× EUCAST tested on isolates treated at 1× EUCAST.

(a) Representative ribosome phenotypes from S2 cells correctly classified as susceptible, showing an elongated phenotype and compact nucleoid; S2 cells incorrectly classified as resistant, showing shorter cells and a less defined nucleoid region; R4 cells incorrectly classified as susceptible, showing a compact central nucleoid; and R4 cells correctly classified as resistant, with diffuse nucleoid regions. Scale bar, 2 µm.

(b) The fraction of cells in the 1× EUCAST sample called Resistant by the 20× EUCAST ciprofloxacin classifier is plotted against the MIC of the strain (mg/L) on a logarithmic scale. Each data point represents a biological replicate. The test dataset is composed of images of S2 and R4 treated with 0.5 mg/L ciprofloxacin for 30 minutes. The EUCAST breakpoint and treatment concentration (0.5 mg/L) is shown with a vertical line. The classifier has high accuracy on R4, but was unable to reliably classify S2.

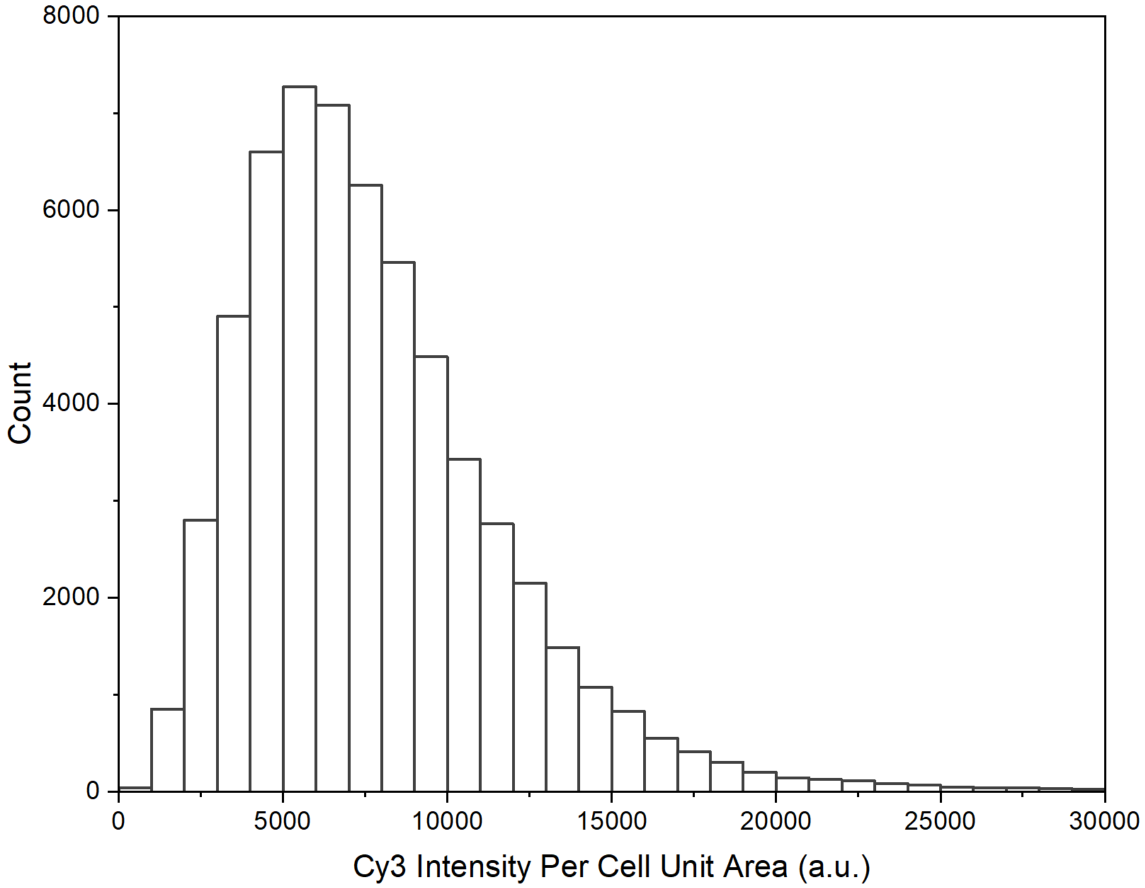

Figure S13. Labelling efficiency of FISH protocol measured by Cy3 intensity. Distribution of cell brightness (Cy3) measured by intensity per cell unit area (bin size = 1000 a.u.). The distribution shows a positive skew and very few cells with low intensity.
